# Searching for Metabolic Markers for Hypertension in Human Urine

**DOI:** 10.3390/molecules31183349

**Published:** 2026-09-21

**Authors:** Adriana Sousa, Nádia Oliveira, Maider Bizkarguenaga, Angela de Diego, Ricardo Conde, Nieves Embade, Óscar Millet, Ignacio Verde

**Affiliations:** 1 Health Sciences Research Centre (CICS-UBI), University of Beira Interior (UBI), 6200-506 Covilhã, Portugal; amssousa@fcsaude.ubi.pt (A.S.); nadiaoliveira@fcsaude.ubi.pt (N.O.); ralves@cicbiogune.es (R.C.); 2 RISE-Health, Faculty of Health Sciences, University of Beira Interior (UBI), Av. Infante D. Henrique, 6200-506 Covilhã, Portugal; 3 Center for Cooperative Research in Biosciences (CIC-BioGUNE), Bizkaia Science and Technology Park, 48160 Derio, Spain; mbizcarguenaga@cicbiogune.es (M.B.); adiego@cicbiogune.es (A.d.D.); nembade@cicbiogune.es (N.E.); omillet@cicbiogune.es (Ó.M.)

**Keywords:** NMR, metabolomics, hypertension

## Abstract

Hypertension is a leading risk factor for the development of cardiovascular diseases and is associated with higher morbidity and mortality rates. However, hypertension screening is not routinely applied to asymptomatic individuals and can be influenced by several factors, such as adequate technique and patient emotional factors, recent exercise, and oral consumptions. Therefore, in this study, we aimed to analyze the urinary metabolomic profiles in normotensive and hypertensive participants by untargeted NMR to identify novel biomarkers for hypertension in a population with a high prevalence of hypertension. Hypertensive participants (when compared with normotensive participants) without cardiovascular or metabolic comorbidities had higher urinary levels of sarcosine and lower levels of acetone, citrate, 4-hydroxyphenylacetate, 3-methylglutaconate, N-isovaleroylglycine, oxaloacetate, phenylalanine and pyridoxate. These metabolites are involved in energy metabolism, amino acid metabolism, gut microbiota-derived metabolism, and vitamin B6 metabolism, reinforcing previous evidence of metabolic changes in hypertension. None of the metabolites were significantly influenced by the presence of cardiovascular or metabolic comorbidities such as dyslipidemia, diabetes, or other cardiovascular diseases, supporting their association with hypertension. To our knowledge, this is the first study to report changes in urinary acetone, oxaloacetate, 3-methylglutaconate, N-isovaleroylglycine and pyridoxate in hypertensive individuals (vs. normotensive individuals). Citrate, methylglutaconate, N-isovaleroylglycine, oxaloacetate, phenylalanine and sarcosine seem to have a higher potential for reliable hypertension biomarkers, since they were also not significantly influenced by any of the anti-hypertensive treatments. Together, these findings expand the current urinary metabolomic profile of hypertension and highlight the potential of these urinary metabolites as a non-invasive approach for earlier hypertension detection.

## 1. Introduction

Hypertension, characterized by chronically elevated blood pressure, is a major risk factor for the development of cardiovascular diseases, and is associated with higher morbidity and mortality rates [1]. Therefore, early detection, monitoring, and treatment of this condition are crucial for the prognosis of affected individuals.

Even though blood pressure measurement is an accessible method to perform, it is not routinely applied to all asymptomatic individuals. According to the latest report from WHO, 46% of hypertensive people 30–79 years old are not diagnosed and are completely unaware of their condition. Furthermore, blood pressure measurement is also influenced by technique and the patient’s emotional state (stress, white-coat syndrome) and consumptions (caffeine, tobacco, energy beverages, etc.) [2]. Also, some pathological changes are already occurring before blood pressure rises [1].

Previous studies have focused on the metabolomics of hypertension, highlighting the possible connection between this condition and intestinal dysbiosis [2,3,4,5,6,7,8], inflammation and oxidative stress [9,10,11,12,13], glucose metabolism and insulin resistance [14], and glycolysis [11,13,15,16]. However, most studies focused on blood samples (serum or plasma), while urine-based research is still scarce.

Urine metabolomics is particularly important for complementing blood-based studies, not only because its collection is much simpler and non-invasive, but also because it detects metabolites excreted by the kidney, reflecting downstream metabolism [17]. Although excreted metabolites suffer more variations due to hydration and diet, this variability makes it possible to closely monitor metabolic changes, disease progression and downstream effects of hypertension, and can provide insights into the influence of gut microbiota in metabolites [17,18].

Previous studies of urine from individuals with hypertension have identified changes in metabolites involved in energy, amino acid, gut microbiota-derived and neurotransmitter/steroid hormone metabolisms, and renal function and salt handling. Energy metabolism seems to be one of the most implicated metabolisms, with several reported urinary changes, such as higher levels of 5-hydroxyhexadecanoate in individuals with hypertension vs. normotensive [19], an inverse association between urinary citrate excretion and prevalent hypertension [20], and a positive association between alanine excretion levels in 24 h urine samples with blood pressure [4]. Higher urinary levels of citrate and oxaloacetate have also been reported in individuals with resistant hypertension when compared with individuals with controlled hypertension [21]. Regarding amino acid metabolism, higher levels of o-tyrosine as well as lower levels of L-methionine and 2-aminooctanoate have been reported in individuals with hypertension (vs. normotensive). As mentioned above, intestinal dysbiosis and hypertension seem to be connected: other authors have observed a positive association between formate excretion levels in 24h urine samples and blood pressure and an inverse association between hippurate excretion levels and blood pressure [4], and higher levels of butyrate in individuals with hypertension [19]. Regarding neurotransmitter and steroid hormone metabolism, Zhao et al. observed higher levels of urinary cortolone, 11-hydroxyandrosterone, and lower levels of melatonin, 3,4-dihydroxyphenylglycol, and 5-hydroxyindolacetate in individuals with hypertension vs. normotensive [19]. Renal function and salt handling also seem to be altered: Matafora et al. found higher levels of uromodulin in proteomic analysis of urine from hypertensive vs. normotensive individuals (and higher uromodulin levels were also associated with decreased levels of salt excretion), and an association between urinary nephrin 1 (a marker of glomerular slit diaphragm) and salt-sensitive individuals (and a positive correlation with increased albuminuria) [22].

In this study, our goal was to analyze the urine metabolomic profiles of normotensive and hypertensive participants by using untargeted NMR to identify novel biomarkers for hypertension in a population with a high prevalence of hypertension.

## 2. Results

### 2.1. Sociodemographic and Clinical Data

Table 1 and Table 2 summarize the sociodemographic characteristics and health status of the different groups of participants. The normotensive (NT) group was formed of participants without a hypertension diagnosis. Participants with hypertension (HT) formed the HTall group, and participants with hypertension but without any other cardiovascular and/or metabolic comorbidity formed the HTo subgroup (part of the HTall group). The smaller size of the NT group reflects the high prevalence of hypertension in the studied population and other metabolic and cardiovascular conditions in this age group, and, therefore, the consequent limited availability of controls. Regarding age, we found that the participants in the HTall group were significantly older than those in the NT group. However, when comparing NT participants with those of the HTo group, we did not identify any significant differences (Table 1). As for sex and hypertension diagnosis, although there is a higher percentage of women in the HTall and HTo groups compared with the NT group, the results did not reach statistical significance (Table 1). Body mass index (BMI) mean values corresponded to the “overweight category” (25–29.9 kg/m^2^) in NT, HTo and HTall, and there were no significant differences between the NT group and the HTall or HTo groups (Table 1).

Concerning both systolic and diastolic blood pressures, there were no significant differences between the participants in the HTall or HTo groups vs. the NT group, which can reflect an effective blood pressure control in LTCF through medication and lifestyle changes (Table 1).

As for the biochemistry data, serum levels of triglycerides, total cholesterol, and HDL cholesterol in the NT, HTo and HTall groups were considered normal. However, glucose values were slightly above normal range in the HTall group, but normal in the NT and HTo groups. Also, LDL cholesterol values were elevated in the NT and HTo groups, but normal in the HTall group (Table 1). When comparing the NT group with the HTo and HTall groups, we did not find any differences in triglycerides, glucose, or HDL cholesterol levels. On the other hand, we found that the levels of LDL and total cholesterol were significantly lower in the HTall group compared with the NT group, which can point to the fact that individuals presenting several comorbidities are being properly monitored and receiving adequate treatment.

Regarding cognitive evaluation, although there were no significant differences in the average ACE-R and GDS scores between groups, we found significantly lower scores for MMSE in participants in the HTall group (vs. NT), and especially in participants in the HTo group (Table 1).

Table 2 shows the percentage of use of different classes of drugs in the NT, HTall and HTo groups. As expected, there was a significantly higher use of angiotensin-receptor antagonists (ARA) and diuretics in both the HTall group and the HTo subgroup vs. the NT group, and a higher use of AnCoa therapy in the HTall group vs. the NT group (Table 2). Other anti-hypertensive and cardiovascular drugs were also more widely used in the HTall group (vs. NT group), such as antianginal, ACEi, BAA, CCB, and venotropic drugs, but did not achieve statistical significance (Table 2). Regarding drug treatments for metabolic, respiratory, and central nervous system conditions, we did not observe any significant differences between the NT and HTall or HTo groups (Table 2).

### 2.2. Univariate Analysis of Metabolites

**Figure 1:**
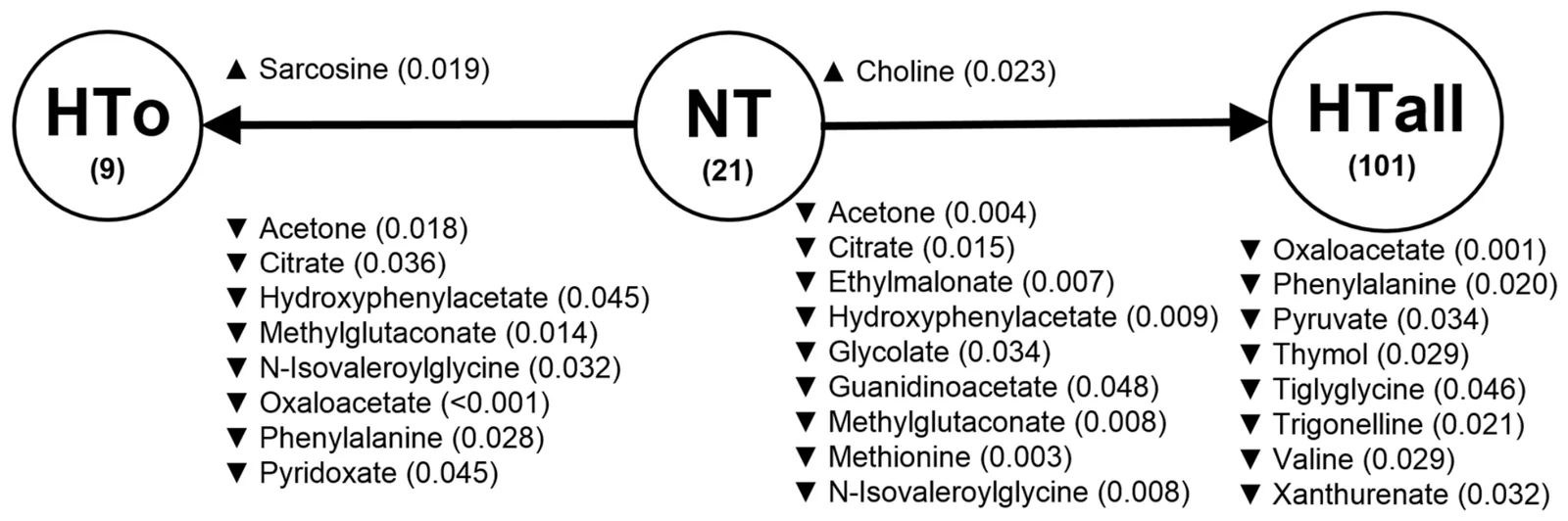
Summary of changes in metabolite levels observed between the NT group and the HTo (**left**) and HTall (**right**) groups. The symbols indicate an increase (▲) or decrease (▼) in urinary metabolite levels in the HTo or HTall vs. the NT group; the number between parenthesis indicates the *p*-value. In the circles of each group/subgroup, we indicated in parenthesis the number (*n*) of participants in each group/subgroup. Statistical analysis was performed using Student’s *t*-test or Mann–Whitney test.

**Figure 2:**
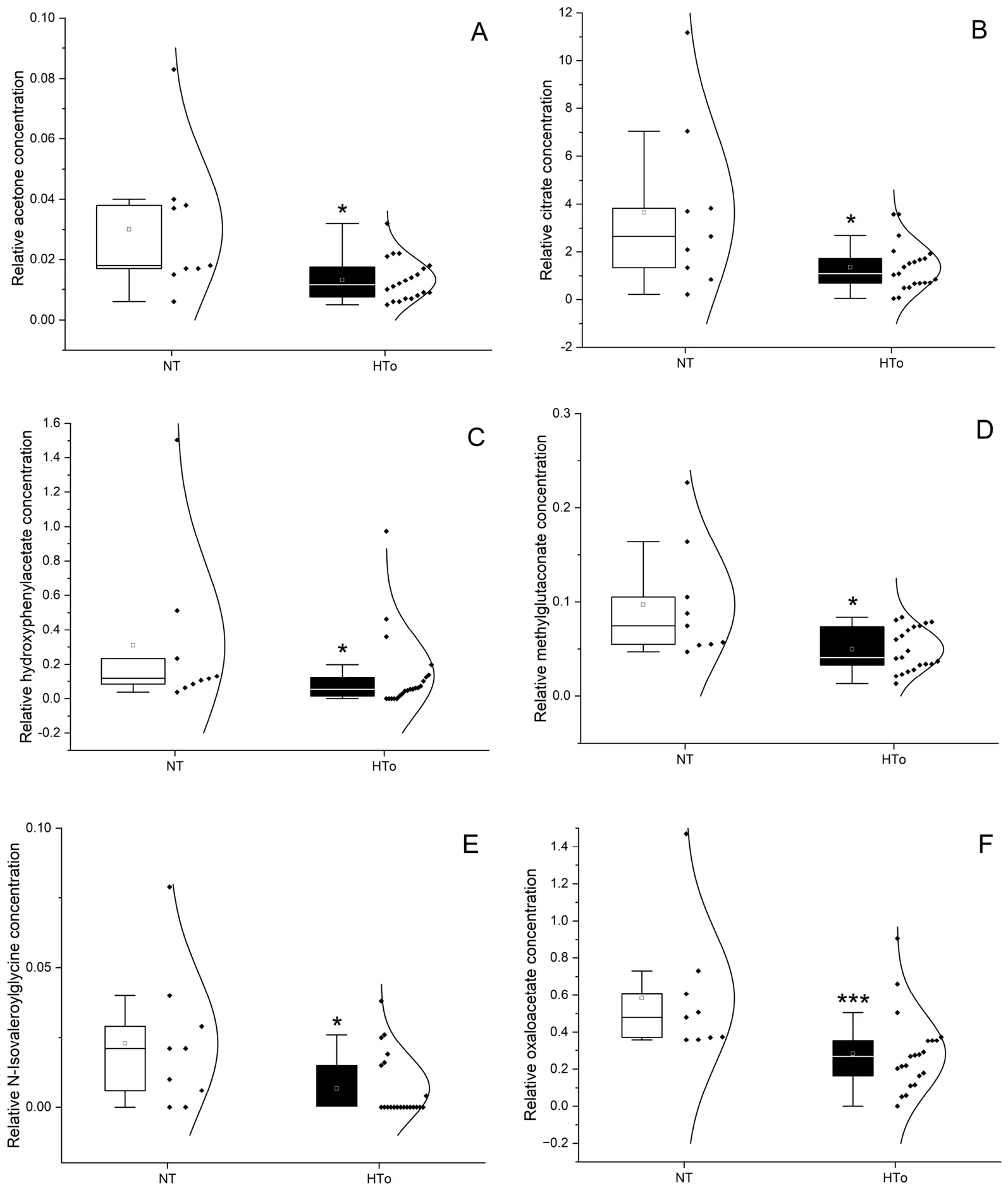

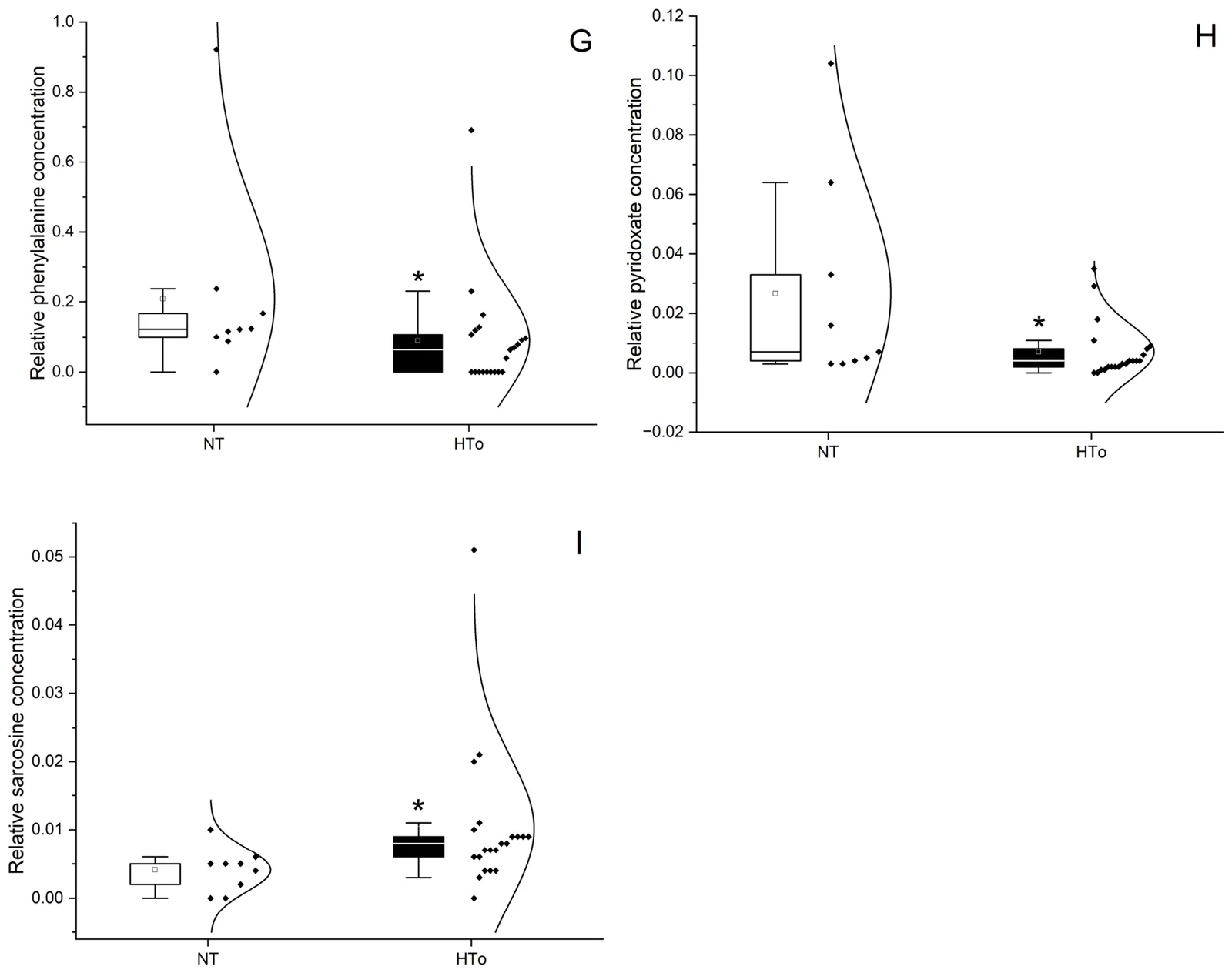
Violin plots representing the distribution of metabolite concentrations (mmol/L) for the metabolites that were significantly different between the NT group vs. the HTo subgroup. (**A**) acetone; (**B**) citrate; (**C**) 4-hydroxyphenylacetate; (**D**) methylglutaconate; (**E**) N-isovaleroylglycine; (**F**) oxaloacetate; (**G**) phenylalanine; (**H**) pyridoxate; (**I**) sarcosine. Each dot represents each participant’s urine sample. The width of the violin plot indicates the density of the data at each concentration. Statistical analysis was performed using Student *t*-test (STT). * *p* < 0.05, *** *p* < 0.001 vs. NT group.

To understand the influence of blood pressure and hypertension on urine metabolites, we performed an untargeted urine metabolite analysis and compared the metabolite concentrations of the HTo subgroup and the NT group. We found higher levels of sarcosine, and lower levels of acetone, citrate, 4-hydroxyphenylacetate (4-HPAA), methylglutaconate, N-isovaleroylglycine, oxaloacetate, phenylalanine, and pyridoxate, in the urine of participants in the HTo group (Figure 1 and Figure 2, and Supplementary Table S1). In the HTall group, we also found a congruent decrease in almost all of these metabolites: acetone, citrate, 4-HPAA, methylglutaconate, N-isovaleroylglycine, oxaloacetate, and phenylalanine; however, there was no difference in pyridoxate and sarcosine levels (vs. NT). Regarding other metabolites in the HTall group (vs. NT), we also found a significant increase in choline, and a decrease in ethylmalonate, glycolate, guanidinoacetate, methionine, pyruvate, thymol, tiglyglycine, trigonelline, valine and xanthenurate (Figure 1 and Supplementary Table S2).

Then, to understand the possible confounding effects of other comorbidities, we subdivided hypertensive participants from the HTall group into smaller subgroups, according to their comorbidities, such as dyslipidemia, diabetes, or other cardiovascular diseases: The HTdl subgroup was made up of hypertensive individuals diagnosed with dyslipidemia but without diabetes or cardiac diseases; the HTdb group was composed of hypertensive individuals diagnosed with diabetes but without dyslipidemia or any other cardiac diseases; the HTcd subgroup comprised hypertensive individuals diagnosed with one cardiac disease (heart failure, arrhythmia, or history of acute myocardial infarction) but without diabetes or dyslipidemia; the HTdb•dl subgroup was composed of hypertensive individuals diagnosed with dyslipidemia and diabetes but without any other cardiac disease diagnosed; the HTcd•dl subgroup was made up of hypertensive individuals diagnosed with dyslipidemia, and only one cardiac disease; and the HTcd•db subgroup comprised hypertensive individuals diagnosed with diabetes and one of the cardiac diseases mentioned earlier.

**Figure 3:**
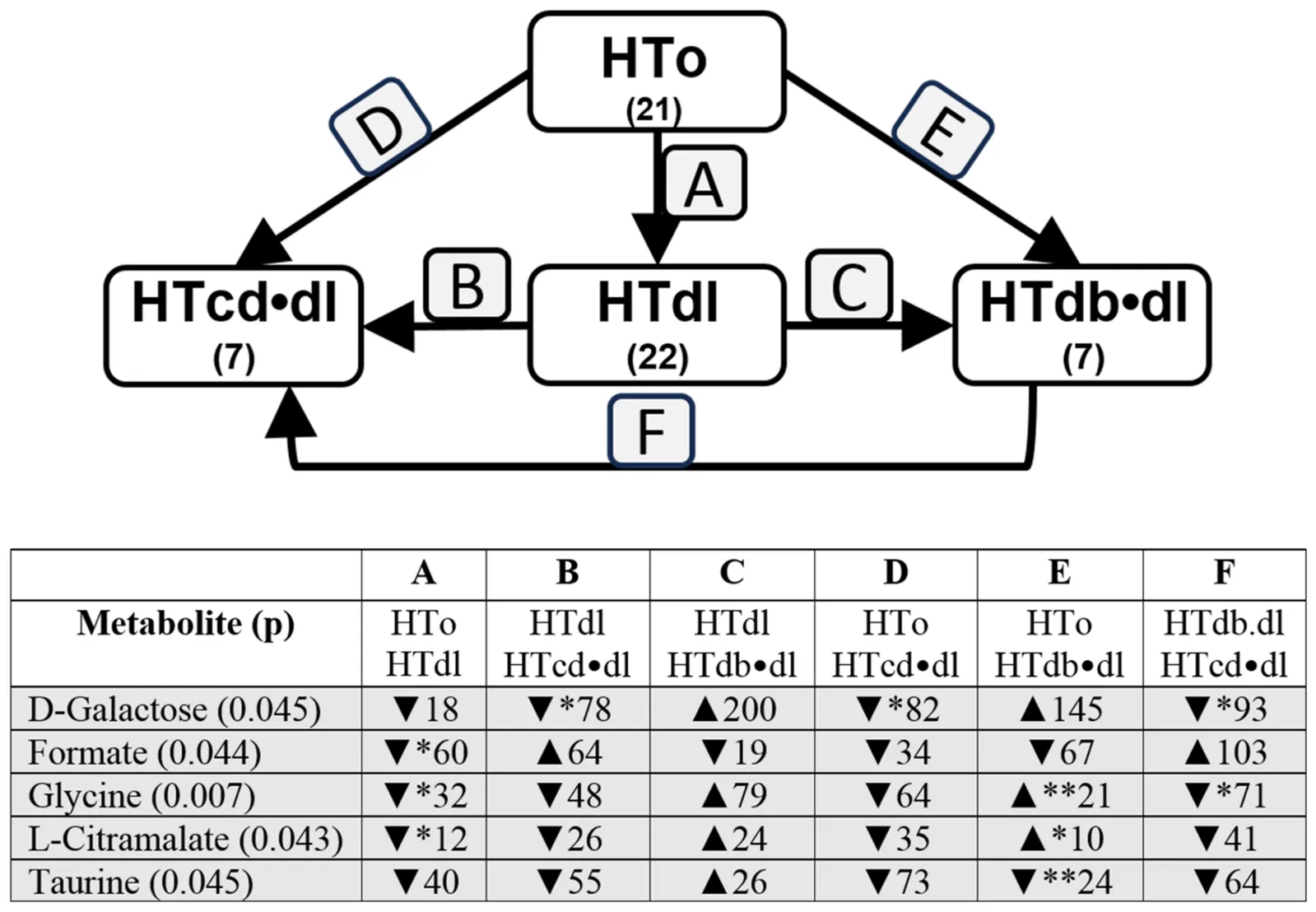
Comparison of urinary metabolite levels between the HTo subgroup and subgroups of hypertensive individuals with dyslipidemia (with or without other comorbidities): hypertensive individuals with dyslipidemia only (HTdl), hypertensive individuals with dyslipidemia and diabetes (HTdb•dl) and hypertensive individuals with dyslipidemia and one cardiac disease (HTcd•dl). Comparison was performed between two subgroups at a time using Kruskal–Wallis test (KWT), followed by post hoc Dunn–Bonferroni Test. The table indicates all the metabolites that had a significant KWT test result (*p* < 0.05) and indicates if there was an increase (▲) or decrease (▼) in each metabolite and the percentage of change relative to the first mentioned subgroup. * *p* < 0.05; ** *p* < 0.01.

Thus, Figure 3 and Supplementary Table S3 show changes in some metabolites in the dyslipidemia subgroups (HTdl, HTdb•dl and HTcd•dl). In the HTdl vs. HTo analysis, we found significant lower levels of formate, glycine, and L-citramalate. When comparing participants with dyslipidemia plus diabetes (HTdb•dl subgroup) with the NT group, we found a significant increase in glycine and L-citramalate, and a decrease in taurine. We found significantly lower levels of D-galactose in participants with dyslipidemia plus one other cardiovascular disease (HTcd•dl) vs. HTo. We also compared metabolites between the HTdl subgroup and the other subgroups, and found that, in the HTcd•dl group, participants had lower levels of D-galactose, while in the HTdb•dl group, despite a significant increase, there were no significant differences. Lastly, when comparing HTdb•dl vs. HTcd•dl, we found lower D-galactose and glycine levels in the latter group. Thus, none of the metabolites that were significantly altered in the HTall group or HTo subgroup vs. the NT group had any significant differences in this analysis with respect to dyslipidemia comorbidity.

**Figure 4:**
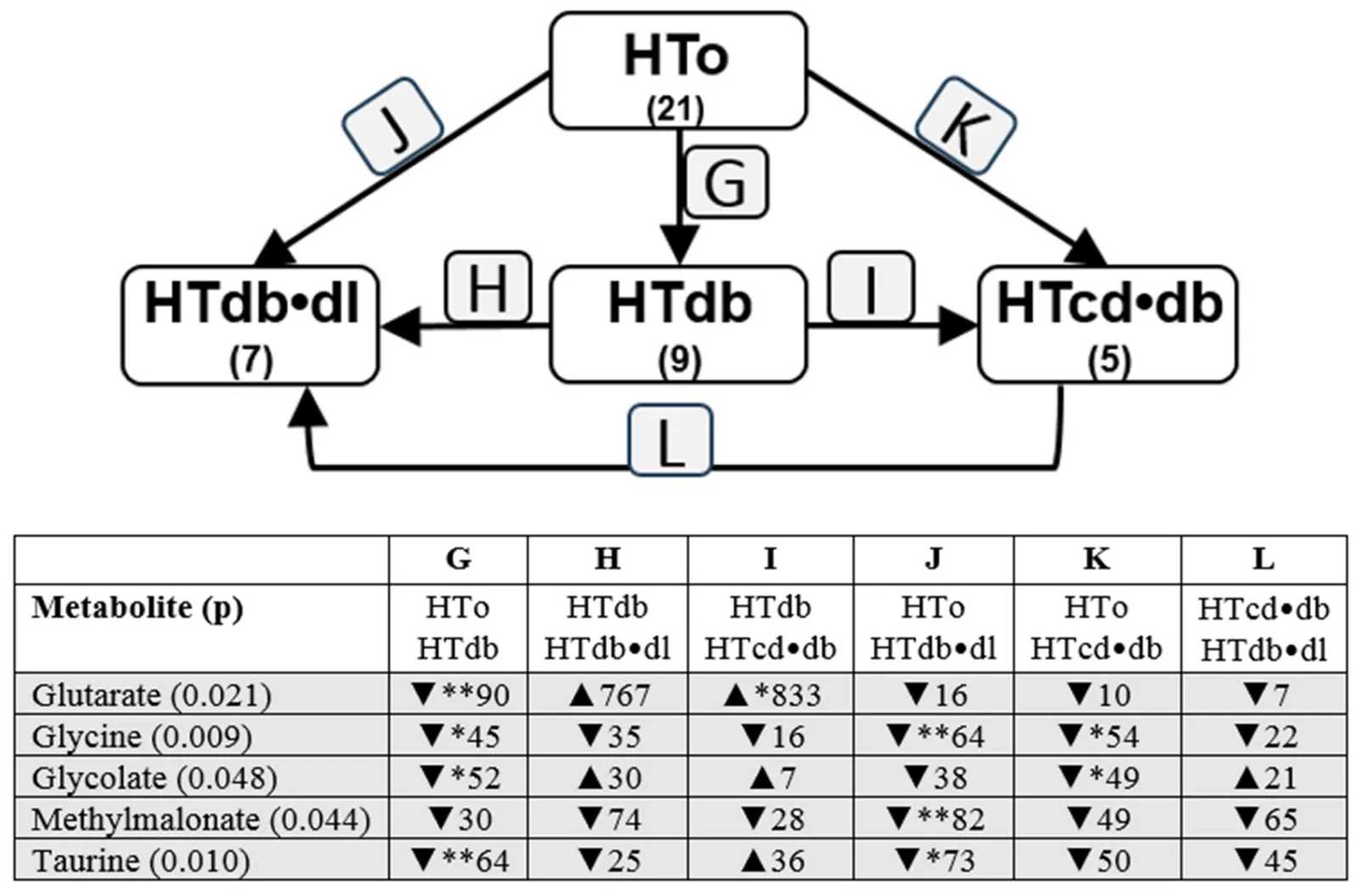
Comparison of urinary metabolite levels between the HTo subgroup and subgroups of hypertensive individuals with diabetes (with or without other comorbidities): hypertensive individuals with diabetes only (HTdb), hypertensive participants with diabetes and dyslipidemia (HTdb•dl), and hypertensive participants with diabetes and one cardiac disease (HTcd•db). Comparison was performed between two subgroups at a time using Kruskal–Wallis test (KWT), followed by post hoc Dunn–Bonferroni test. The table indicates all the metabolites that had a significant KWT test result (*p* < 0.05) and if there was an increase (▲) or decrease (▼) in each metabolite and the percentage of change relative to the first mentioned subgroup. * *p* < 0.05; ** *p* < 0.01.

Figure 4 and Supplementary Table S4 show changes in metabolites between the diabetes subgroups (HTdb, HTcd•db, and and HTdb•dl). Compared with the HTo group, participants in the HTdb subgroup had significantly lower levels of glutarate, glycine, glycolate, and taurine. When comparing participants that had diabetes plus dyslipidemia with the HTo group, we found lower levels of glycine, methylmalonate, and taurine in the HTdb•dl subgroup. The comparison of participants with one cardiovascular disease plus dyslipidemia with HTo revealed the existence of lower levels of glycine and glycolate in HTcd•db subgroup. Then, when comparing HTdb with other subgroups, we found significantly higher levels of glutarate in the HTcd•db subgroup. Lastly, we did not find any significant differences in metabolite levels between HTdb•dl and HTdb, or between HTcd•db and HTdb•dl. Glycolate, which was significantly decreased in the HTall group (vs. NT), was, as mentioned, significantly decreased in HTdb and HTcd•bd subgroups vs. HTo. This can suggest that the decrease of glycolate in the HTall could be influenced by the presence of diabetes and/or cardiac diseases in hypertensive individuals, since urinary glycolate levels were not significantly different when we compared NT and HTo groups.

**Figure 5:**
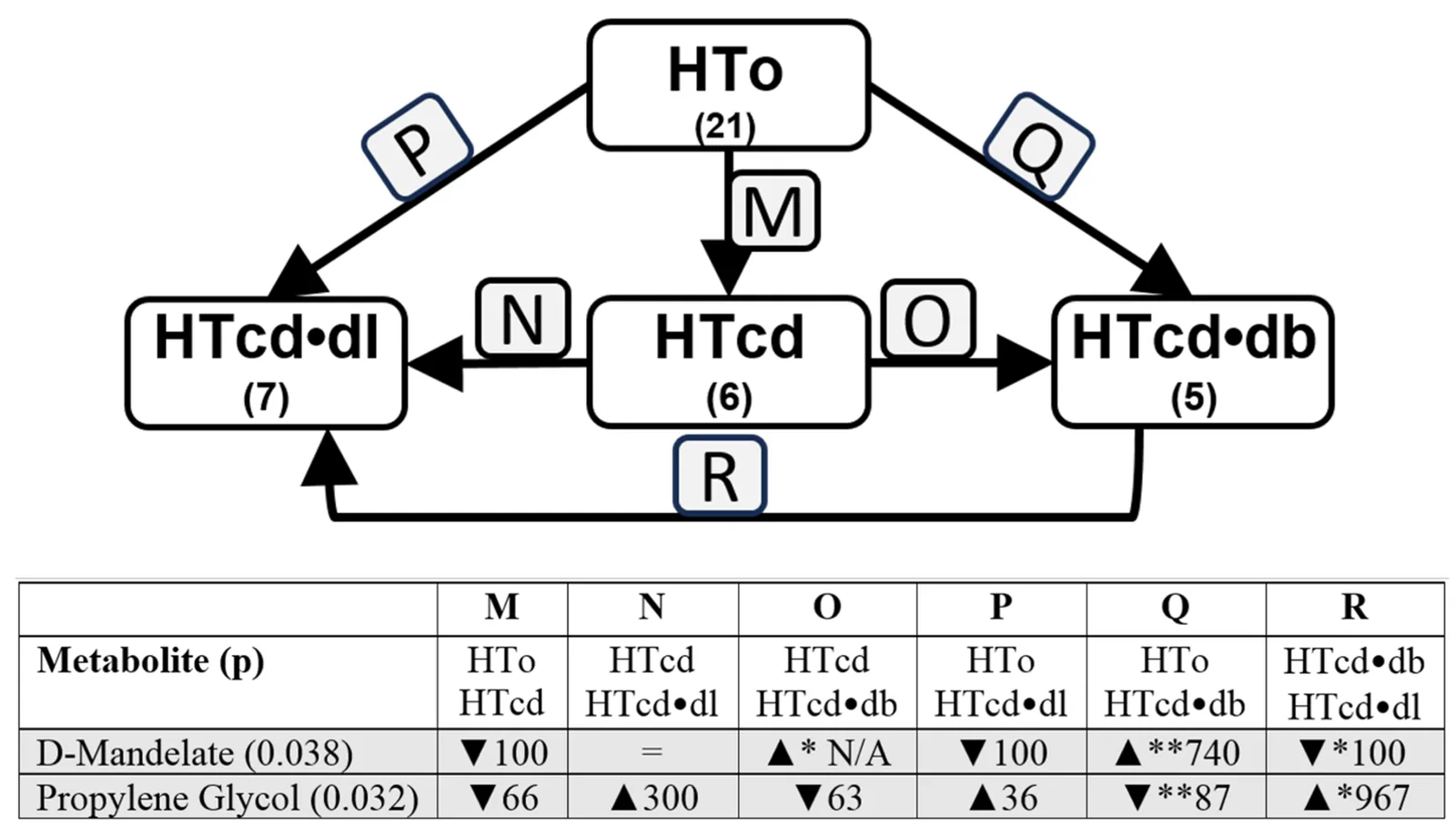
Comparison of urinary metabolite levels between the HTo subgroup and subgroups of hypertensive individuals with one cardiac disease (with or without other comorbidities): hypertensive individuals with one cardiac disease only (HTcd), hypertensive individuals with one cardiac disease and dyslipidemia disease (HTcd•dl) and hypertensive individuals with one cardiac disease and diabetes (HTcd•db). Comparison was performed between two subgroups at a time using Kruskal–Wallis test (KWT), followed by post hoc Dunn–Bonferroni test. The table indicates all the metabolites that had a significant KWT test result (*p* < 0.05) and if there was an increase (▲) or decrease (▼) in each metabolite and the percentage of change relative to the first mentioned subgroup. * *p* < 0.05; ** *p* < 0.01. N/A: percentage of change not applicable (baseline value = 0).

Figure 5 and Supplementary Table S5 show changes in some metabolites between the cardiovascular subgroups (HTdb, HTcd•dl, and HTcd•db) and HTo. We found significantly higher levels of D-mandelate in the HTcd•db subgroup when compared with HTo, HTcd and HTcd•dl. Also, propylene glycol levels were significantly lower in HTcd•db when compared with HTo and HTcd•dl.

None of the metabolites that were significantly different between individuals in the HTo and NT groups were significantly influenced by diabetes, dyslipidemia or cardiac diseases. Among the metabolites that were significantly different between HTall and NT groups, only glutarate was significantly influenced by diabetes; all other metabolites were not significantly influenced by any of the comorbidities.

**Figure 6:**
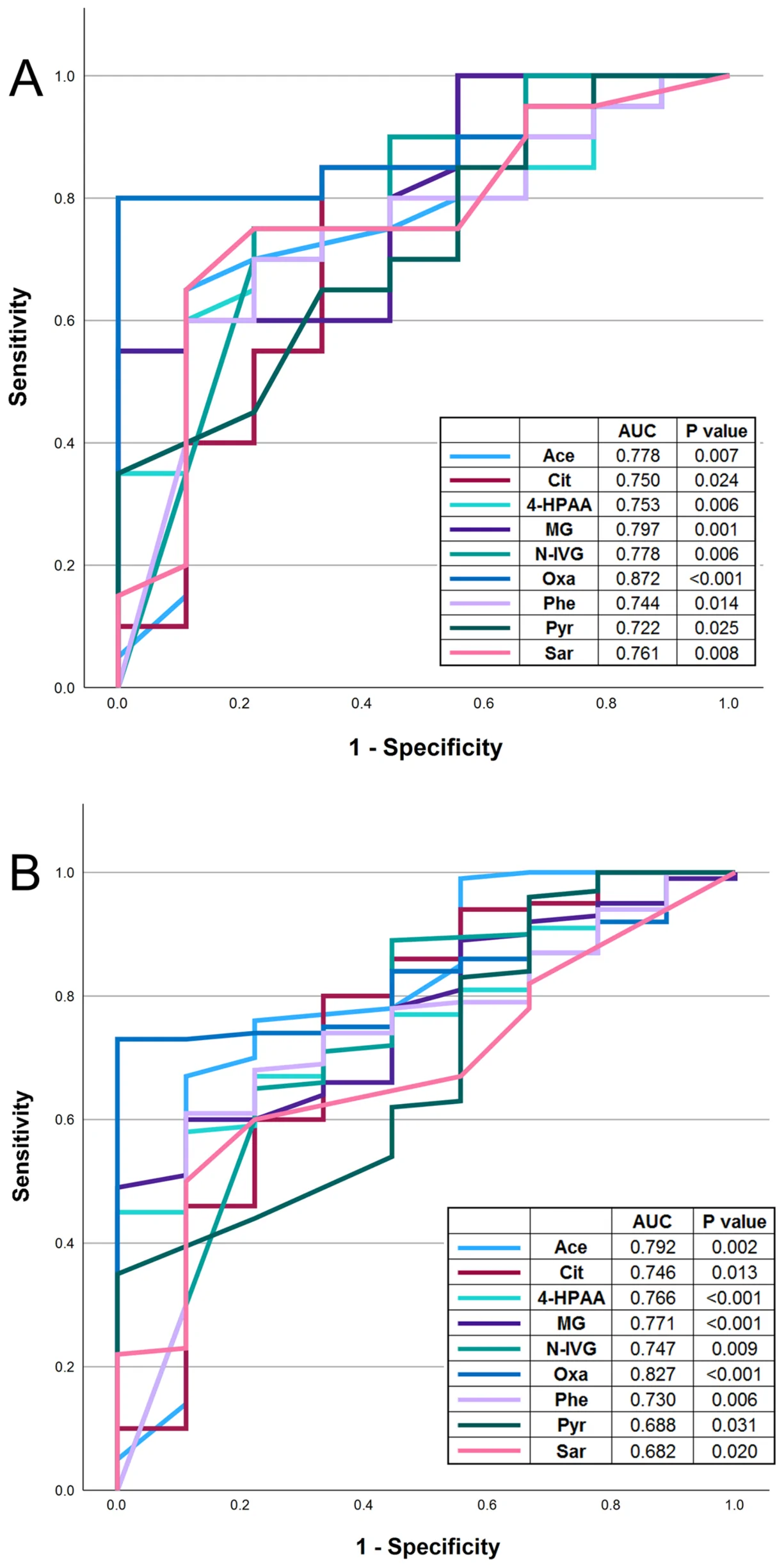
AUROC analysis to discriminate HTo (**A**) and HTall (**B**) from NT based on the urinary concentrations of the following metabolites: acetone (Ace), citrate (Cit), 4-hydroxyphenylacetate (4-HPAA), 3-methylglutaconate (MG), N-isovaleroylglycine (N-IVG), oxaloacetate (Oxa), phenylalanine (Phe), pyridoxate (Pyr), and sarcosine (Sar).

The discriminatory power of the nine metabolites that showed significant differences between HTo and NT for hypertension was estimated from the analysis of the area under the receiver operating characteristic curve (AUROC; sensitivity/specificity), using the concentrations obtained in the univariate analysis. When this analysis was performed with the NT and HTo groups, all the nine metabolites showed an AUROC equal to or higher than 0.7, with significant *p*-values, indicating good discriminatory power for discriminating hypertension without comorbidities (Figure 6A). When AUROC analysis was performed with the NT and HTall groups, all metabolites except two had AUROC values higher than 0.7 with significant *p*-values, suggesting that these seven metabolites can discriminate hypertension even in the presence of comorbidities (Figure 6B). Pyridoxate and sarcosine had AUROC values of 0.688 and 0.682, respectively, close to 0.7, and both had significant *p*-values, indicating a slightly less discriminant power for hypertension with comorbidities than the other seven metabolites (Figure 6B).

### 2.3. Analysis of Possible Influence of Drug Treatments

As previously mentioned, drug classes such as ARA, DIU and AnCoa were used by a higher percentage of individuals in the HTall group than in the NT group. To understand the possible confounder influence of anti-hypertensive/cardiovascular treatments on metabolite levels, we analyzed whether there were differences in these levels among therapeutic clusters dependent on the anti-hypertensive treatments taken by participants in the HTall group. Since we did not have a large number of participants in the HTall group receiving some monotherapies, we also analyzed several drug combinations of treatments with the following drug classes: angiotensin-converting enzyme inhibitors (ACEi), angiotensin II receptor antagonists (ARA), beta-adrenergic antagonists (BAA), calcium channel blockers (CCB), diuretics (DIU), and anticoagulants (AnCoa). The analysis was performed between clusters of individuals without any of these therapies and individuals with one of these therapies/combinations. Thus, the analyzed clusters were composed of individuals not taking any anti-hypertensive drugs (without drugs; the control set), and individuals treated either with ACEi, ACEi+AnCoa, ACEi+AnCoa+DIU, ACEi+AnCoa+CCB+DIU, ARA, ARA+DIU, ARA+AnCoa+DIU, ARA+AnCoa+CCB, ARA+AnCoa+DIU+CCB, DIU, DIU+AnCoa, or DIU+AnCoa+ARA, or DIU+AnCoa+BAA (Table 3). This analysis was performed for the metabolites that differed significantly between the NT and HTo groups (acetone, citrate, 4-HPAA, methylglutaconate, N-isovaleroylglycine, oxaloacetate, phenylalanine, pyridoxate, and sarcosine) to elucidate if these therapies could influence the significant differences found between the studied groups.

Thus, Table 3 shows the means, standard error, and percentage of change of the urine metabolite levels in each drug cluster when compared with the control cluster. Most comparisons between these clusters did not show significant differences. The significant differences were only found in urinary levels of acetone, 4-HPAA, and pyridoxate.

Regarding urinary levels of acetone, we found that participants treated with ACEi or ARA+AnCoa+CCB had significantly higher levels when compared with individuals not taking treatment. However, individuals taking other drug combinations did not have significantly different acetone levels. We also found significantly higher levels of 4-HPAA in individuals taking ACEi compared to individuals not taking anti-hypertensive treatment, but we did not find other significant differences within other clusters.

Lastly, we found significant changes in urinary pyridoxate levels when comparing individuals treated with some drug combinations and individuals without treatment, such as a significant increase in the cluster ARA+AnCoa+CCB, ARA+DIU and DIU clusters vs. participants not taking treatments.

As for the metabolites citrate, methylglutaconate, N-isovaleroylglycine, oxaloacetate, phenylalanine, and sarcosine, we did not find significant differences among the drug clusters.

## 3. Discussion

Finding novel urinary biomarkers for hypertension would allow for an earlier and more consistent patient diagnosis, leading to earlier access to blood pressure control (through lifestyle changes and drug treatment) and improved prognosis of this disease through management and treatment before irreversible organ damage has occurred. Also, urine can be very easily collected non-invasively by either individuals or their caregivers and it also has the benefit of reflecting downstream metabolism. However, very few studies have focused on searching for hypertension biomarkers in urine samples.

We analyzed data and urine samples from older adults from LTCFs, which allowed us to study the metabolomics of individuals with a high prevalence of hypertension, in a similar age group (>64 years), with similar living conditions, and closely monitored and medicated by the LTCF team, according to their diagnosed diseases. Although we did not identify differences in systolic or diastolic blood pressures between the HTall or HTo groups and the NT group, significant metabolic differences were still expected due to the chronic effects of hypertension.

In urine, we found that individuals with hypertension in the absence of cardiovascular and metabolic comorbidities (vs. normotensive participants) had higher levels of sarcosine and lower levels of acetone, citrate, hydroxyphenylacetate, methylglutaconate, N-isovaleroylglycine, oxaloacetate, phenylalanine, and pyridoxate.

Acetone is one of the three ketone bodies (along with acetoacetate and β-hydroxybutyrate) produced by the liver in ketogenesis, primarily from acetyl-CoA derived from fatty acid β-oxidation. Ketone bodies are used as alternative energy sources, particularly when there is increased fatty acid β-oxidation and acetyl-CoA production under low available glucose conditions, such as during prolonged fasting, carbohydrate restriction, during prolonged exercise, decompensated diabetes (diabetic ketoacidosis), or excessive alcohol intake [23,24]. We found lower urinary acetone levels in hypertensive individuals with and without comorbidities (HTall and HTo, respectively) than in healthy normotensive individuals. The decrease in acetone in urine might indicate possible changes in ketone metabolism and handling. This is the first study to report and compare urinary acetone levels in hypertensive vs. normotensive individuals. Our previous findings with serum samples showed higher glycerol and acetate levels in hypertensive individuals when compared to normotensive individuals [2]. Other authors also found higher levels of acetone in Uygur individuals with hypertension when compared with healthy subjects [25]. To understand the possible confounder effect of dyslipidemia, diabetes, and other cardiac diseases, we analyzed acetone levels between several comorbidity subgroups. We did not find differences in acetone levels between subgroups and individuals diagnosed only with hypertension. The possible confounder effect of anti-hypertensive treatments was also studied between clusters of participants taking different drug combinations. Participants treated with either ACEi or ARA+AnCoa+CCB had significantly higher urinary levels of acetone than those not taking treatments. This indicates that these drugs and drug combinations increase acetone urine levels, influencing acetone’s biomarker potential, since acetone levels are decreased in individuals with hypertension. Thus, our data indicate the decrease in acetone in urine as a possible biomarker of hypertension, even when individuals are taking some drug treatments, such as ACEi or ARA+AnCoa+CCB.

Phenylalanine is an essential aromatic amino acid abundant in dietary protein. It is the precursor of L-tyrosine, which is needed for proteins, catecholamines (adrenaline, noradrenaline, and dopamine) and melanin synthesis [26]. We found lower urinary phenylalanine levels in the HTall group and HTo subgroup than in NT participants. In our previous findings in serum, we did not find differences between hypertensive and normotensive individuals, suggesting that lower urinary phenylalanine levels are not due to changes in its production. Therefore, this decrease could be due to a decreased phenylalanine filtration or increased reabsorption, a decreased gut breakdown of aromatic amino acids by microbiota, or more efficient metabolic use. Other authors have reported conflicted findings regarding the relationship between phenylalanine and blood pressure. Some studies reported no relationship between blood pressure and phenylalanine levels in 24 h urine samples [27] or serum [28]. Other studies found a negative association between phenylalanine plasma levels and diastolic blood pressure in severely frail individuals [29]. Other studies have also reported a relationship between higher phenylalanine intake and higher hypertension risk and blood pressure elevation [30,31,32]. Higher phenylalanine levels have also been associated with age (higher levels were found in centenarians in comparison with other elderly individuals [26]), inflammation [33], and type 2 diabetes [34]. In confounder analysis, we did not find any differences in the comorbidity subgroups or between drug clusters, suggesting that comorbidities such as dyslipidemia, diabetes or cardiovascular diseases and anti-hypertensive treatment do not influence phenylalanine urinary levels. Therefore, the decrease in phenylalanine is a possible biomarker for hypertension, especially since it does not seem to be influenced by other comorbidities or anti-hypertensive drug regimens.

Sarcosine (N-methylglycine) is an amino acid intermediate in the one-carbon and glycine metabolism, formed through glycine methylation. We found higher urinary levels of sarcosine in individuals diagnosed only with hypertension (HTo) than in normotensive participants. Higher sarcosine levels could be due to an increase in renal excretion, a decrease in its recycling into glycine, and/or a decrease in its production (compatible with the decreased glycine levels we found in serum). We also found higher levels of sarcosine in individuals with hypertension and comorbidities (HTall), but without statistical significance. Our previous findings regarding serum metabolites in hypertension [2] have shown lower sarcosine, glycine, and glutamine levels in hypertensive individuals than in normotensive individuals. This difference between urinary and serum sarcosine levels in hypertensive individuals reflects a compartmentalization of this metabolite, from blood to urine. Several authors have also reported altered levels of sarcosine in dyslipidemia [35], pulmonary artery hypertension [36], and prostate cancer [37,38]. We did not find differences between sarcosine levels in comorbidity subgroups and HTo subgroup, or between treated and untreated individuals. This suggests that the increase in sarcosine levels is a potential hypertension biomarker, irrespective of the studied comorbidities and anti-hypertensive drug regimens.

Pyridoxate is the main catabolic product of vitamin B6, and is excreted in the urine [39]. Urinary pyridoxate levels are directly correlated with dietary intake of vitamin B6, responding quickly to its changes, and are correlated with pyridoxate plasmatic levels [39]. We found lower pyridoxate levels in hypertensive participants (in the HTall group and HTo subgroup) than in the NT group. This decrease could be associated with a decrease in vitamin B6 availability (due to lower intake, reduced absorption, etc.), reduced metabolic use of vitamin B6 (for example, in amino acid metabolism), or changes in its renal handling. Other authors found lower levels of vitamin B6 in serum of essential hypertensive individuals than in healthy controls [40]. Higher vitamin B6 intake has been reported to have a beneficial effect on blood pressure regulation, lowering blood pressure by 14/10 mmHg in hypertensive individuals [41,42], and as being negatively associated with hypertension prevalence [42]. We did not find any differences between pyridoxate levels in the comorbidity subgroups and the HTo subgroup, suggesting that dyslipidemia, diabetes, and other cardiac diseases do not seem to influence pyridoxate levels. However, we found an increase in pyridoxate levels in the clusters ARA+AnCoa+CCB, ARA+DIU, and DIU than in individuals not taking anti-hypertensive drugs. Decreased urinary pyridoxate levels seem to be a possible hypertension biomarker that is not influenced by the studied comorbidities, with consideration of the possible confounding effects of several drug combinations (ARA+AnCoa+CCB, DIU, and ARA+DIU).

Citrate is one key intermediate produced in the Krebs cycle, from oxaloacetate and acetyl-CoA, which is essential for energy generation. It is the most abundant organic anion in human urine, and its excretion is strongly determined by the individual’s acid-base status (intracellular pH of renal proximal tubule cells) [43]. In acidic conditions, citrate reabsorption by the proximal tubule increases, and its production decreases; in alkaline conditions, the inverse events occur [43]. We found lower urinary citrate levels in individuals with hypertension (with and without comorbidities) than in normotensive individuals. Our previous findings in serum did not show significant changes in citrate levels between hypertensive and normotensive individuals. Other authors have reported an inverse association between urinary citrate excretion and prevalent hypertension, independent of age, body size, and diet [20]. Changes in the citric acid pathway in individuals with resistant hypertension have also been reported, namely, higher urinary citrate and oxaloacetate levels in these individuals than in individuals with controlled hypertension [21]. Low citraturia is also a known risk factor for nephrolithiasis and has been associated with distal renal tubular acidosis, chronic diarrheal states, excessive animal protein consumption, thiazide-induced hypokalemia, and vigorous exercise [43]. We did not find differences in citrate levels between the comorbidity subgroup and the HTo subgroup. We also did not find differences across drug sets, suggesting that these anti-hypertensive treatments do not influence citrate levels. Overall, lower urinary citrate levels seem to be a good hypertension biomarker, especially as they are not influenced by the studied comorbidities and drug sets.

Oxaloacetate is a metabolic intermediate in several biochemical pathways, such as the citric acid cycle, and is involved in excitatory metabolite synthesis. In the citric acid cycle, oxaloacetate is combined with acetyl-coA to form citrate. Oxaloacetate can also be converted to aspartate (through transamination), which can combine with citrulline (in the urea cycle) and generate arginosuccinase, and then L-arginine [44]. L-arginine is a substrate for nitric oxide synthase, generating nitric oxide and citrulline [44]. The generated nitric oxide can have a protective effect against hypertension development through its vasodilation effect, as well as by directly inhibiting sodium reabsorption in nephron segments [44]. We found lower oxaloacetate levels in individuals with hypertension with and without comorbidities (HTall and HTo) than in normotensive individuals. This is the first study reporting urinary oxaloacetate concentration differences between normotensive and hypertensive individuals. Other authors have reported higher oxaloacetate levels in individuals with resistant hypertension than in individuals with controlled hypertension [21]. Santiago Hernandez et al. found lower urinary levels of oxaloacetate in hypertensive individuals with microalbuminuria vs. individuals with normoalbuminuria [45]. We did not find differences across the different comorbidity subgroups and drug sets. Therefore, the decrease in urinary oxaloacetate levels could be a possible hypertension biomarker, especially as it does not seem to be influenced by comorbidities or drug treatments.

3-Methylglutaconate is a branched-chain organic acid involved in mitochondrial leucine catabolism and is found in trace amounts in the urine of healthy individuals [46]. Higher urinary and serum concentrations of this metabolite are usually associated with 3-methylglutaconic acidurias, metabolic conditions caused by specific enzyme deficiencies, or mitochondrial dysfunction, leading to the accumulation of 3-Methylglutaconate and resulting in metabolic acidosis and neurological and developmental abnormalities [46]. We found lower urinary 3-Methylglutaconate levels in hypertensive individuals in the HTall group and HTo subgroup than in normotensive individuals. This decrease could be explained by a decrease in leucine catabolism or amino acid oxidation in general, a change in the substrates used by mitochondria, or increased Krebs cycle efficiency (leading to fewer wasted intermediates). This is the first study to report lower 3-Methylglutaconate levels in the urine of hypertensive individuals. 3-Methylglutaconate urinary levels were not influenced by the presence of comorbidities or different drug treatments. Thus, decreased levels of 3-Methylglutaconate seem to be a potential hypertension biomarker, which is not influenced by comorbidities or drug treatments.

N-Isovaleroylglycine is also a byproduct of leucine catabolism. It is highly increased in individuals with isovaleric acidemia, an autosomal recessive disorder of leucine catabolism, caused by a deficiency of isovaleryl-CoA dehydrogenase, leading to an accumulation of isovaleroy-CoA metabolites [47]. Increased N-isovaleroylglycine levels have also been associated with pediatric nocturnal enuresis [48]. We found lower levels of N-isovaleroylglycine in participants with hypertension (with and without comorbidities) than in normotensive participants. Similarly to 3-Methylglutaconate, decreased levels of this metabolite might be associated with decreased leucine catabolism or reduced amino acid oxidation in general or a change in the substrates used by mitochondria. In the analysis of the comorbidity confounder effect, we did not find significant differences between the comorbidity subgroups and hypertension-only subgroup. Regarding drug treatments, we did not find differences across the different drug sets, suggesting that N-Isovaleroylglycine levels are not influenced by the studied anti-hypertensive drugs. This highlights the potential use of N-Isovaleroylglycine as a hypertension biomarker, irrespective of the comorbidities or drug treatments.

4-Hydroxyphenylacetate (4-HPAA) is an aromatic organic acid derived from tyrosine metabolism, mainly produced by gut microbiota, and which can be absorbed to the bloodstream. Its urinary levels can provide insight into aromatic amino acid metabolism and microbial activity. We found lower urinary levels of 4-HPAA in hypertensive participants (with and without comorbidities) vs. normotensive participants. Lower urine levels of 4-HPAA in hypertension could be associated with reduced microbial aromatic amino acid fermentation, reduced substrate availability (tyrosine), shifts in microbial metabolic pathways, or a systemic metabolic shift. Other authors also reported an inverse association between dietary intake of phenolic compounds and hydroxyphenylacetate and hypertension diagnosis [49]. We did not find significant differences in 4-HPAA urinary levels between the comorbidity subgroups and the hypertension-only subgroup. Higher levels of 4-HPAA were found in individuals taking ACEi, suggesting that this treatment could influence 4-HPAA levels. We did not find differences regarding the other drug combinations. Therefore, 4-HPAA could be a potential hypertension biomarker that is not influenced by comorbidities, with caution regarding the possible confounding effect of ACEi treatment.

Overall, our study indicates changes in several metabolic pathways associated with hypertension, such as energy metabolism (lower urinary levels of acetone, citrate, oxaloacetate, and 3-methylglutaconate), amino acid metabolism (lower urinary levels of phenylalanine and N-isovaleroylglycine, and higher levels of sarcosine), gut microbiota-derived metabolism (lower urinary levels of 4-HPAA), and vitamin metabolism (lower urinary levels of pyridoxate). These results reinforce previous evidence about the association between hypertension and energy, amino acid and B6 vitamin metabolism, and intestinal dysbiosis. This study is also, to our knowledge, the first to report changes in urinary acetone, oxaloacetate, 3-methylglutaconate, N-isovaleroylglycine, and pyridoxate levels in hypertensive individuals. Citrate, methylglutaconate, N-isovaleroylglycine, oxaloacetate, phenylalanine and sarcosine seem to have a higher potential for reliable hypertension biomarkers, since they were not significantly influenced by any of the studied comorbidities or drug treatments. These findings support the potential to use these urinary biomarkers as a non-invasive approach for earlier hypertension detection.

## 4. Methods

### 4.1. Population, Data, and Groups

This study focused on individuals from EBIcohort (Elders from Beira Interior cohort): users of eighteen long-term care facilities (LTCF) from three Portuguese municipalities (Fundão, Covilhã and Belmonte) older than 64 years, covering an area of ~1000 square kilometers. The procedures executed in this study were reviewed and approved by the Ethics Committee of the University of Beira Interior in accordance with the Declaration of Helsinki (Ref. Number CE-UBI-Pj-2017-012). The participants or their legal representatives signed a written informed consent form before starting the study.

We applied the following exclusion criteria for the EBIcohort individuals: (1) Global Deterioration Scale ≥ 6; (2) diagnosis or history of cancer or tumor malignancy in the previous 5 years; (3) diagnosis of serious psychiatric disorders; (4) use of persistent anticonvulsant therapy; (5) presence of several hematologic disorders with abnormalities in the proportion of blood cells; and (6) long-term treatment with antiretroviral therapy.

Initially, we divided the individuals into two main groups based on the presence or absence of hypertension diagnosis: the normotensive group (NT) and the hypertensive group (HTall). Individuals in the NT group did not have any cardiovascular or metabolic diseases (CVMD), such as chronic heart failure, angina pectoris, atrial fibrillation, history of acute myocardial infarction, diabetes, or dyslipidemia. The HTall group included individuals that could have other CVMD.

To perform further analysis of the influence of other cardiovascular or metabolic diseases (hypertension comorbidities), the HTall group was further subdivided into subgroups based on the diagnosis information. Thus, the HTo subgroup was composed of individuals with HT without diagnosis of any other CVMD; HTdl was composed of individuals with only HT and dyslipidemia diagnosis; HTdb was made up of individuals with only HT and diabetes; HTcd comprised individuals with HT and diagnosis of one cardiac disease (either chronic heart failure, angina, arrhythmia, or history of acute myocardial infarction), but not other CVMD; HTdl•db was formed of individuals with HT and further diagnosis of dyslipidemia and diabetes; and HTdl•cd was composed of individuals with HT and diagnosis of dyslipidemia and only one of the above mentioned cardiac diseases.

### 4.2. Data and Sample Collection and Processing

Data concerning the sociodemographic and clinical characteristics and pharmacological treatment of each participant were obtained from the individual and/or clinical staff of the collaborative LTCF. Body mass index (BMI; kg/m^2^) was calculated using height and body weight data. Blood pressure (BP) was measured in the same week as urine and data collection in the LTCF. All the measurements were performed under standard conditions, using a manual or automated sphygmomanometer, which is the gold standard method to measure BP. Systolic Blood Pressure (SBP) and Diastolic Blood Pressure (DBP) results were expressed in mmHg.

The cognitive status of the participants was assessed by a competent team from our group through the application of the Global Deterioration Scale (GDS) and the Addenbrooke’s Cognitive Examination-Revised (ACE-R) test. The GDS scale was developed to evaluate primary degenerative dementia, measuring cognitive decline over time in seven different progressing stages, considering the clinical characteristics and memory deterioration of the individuals The ACE-R is a rapid test battery, with good sensitivity and specificity, to screen for early cognitive impairment and dementia, which can be used to distinguish between frontotemporal dementia and Alzheimer’s disease [50]. It evaluates six cognitive domains: attention/orientation, memory, fluency, language, and visuospatial ability, two of which are part of the Mini-Mental State Examination (MMSE) [50]. This test was already validated for the Portuguese population [51]. MMSE is shorter and also provides rapid screening of cognitive state, distinguishing between normal cognition and mild, moderate, and severe cognitive impairment.

### 4.3. Sample Collection, Transport, and Preparation

We collected urine samples from all the participants, within the same week as data collection, cognitive evaluation, and blood pressure measurement.

First-morning urine was collected by the participants or the accompanying carer/nurse in a sterile urine container, after discarding the first drops. The samples were transported to the laboratory at 4 °C. They were then centrifuged at 6000 rpm for 5 min at 4 °C, separated into 1 mL aliquots, and cryopreserved (−80 °C) until further NMR processing.

### 4.4. NMR Data Acquisition, Processing, and Quantification

Untargeted NMR measurements were performed in a 600 MHz IVDr NMR spectrometer (Bruker BioSpin GmbH, Rheinstetten, Germany) with a tempered SampleJet automatic sample changer and a double=resonance broadband probe (BBI) head with a z-gradient coil and BOSS-III shim system. NMR sample tubes were stored inside the SampleJet at 5 °C until measurement.

According to the standardized Bruker IVDr urine preparation protocol, urine was mixed at a 9:1 (*v*/*v*) ratio with the Bruker IVDr urine phosphate buffer prepared in D_2_O.

Every morning, the spectrometer was calibrated with three different samples, following strict standard operation procedures, as previously described [52]: a methanol reference sample to verify probe temperature and ensure acquisition at 300 K, a QuantRef sample to assess quantitative performance and instrument response, and a sucrose reference sample to evaluate magnetic-field homogeneity, line shape, shimming performance, and overall spectral quality. Study samples were analyzed only after the predefined instrument-performance criteria had been fulfilled. These daily reference measurements also provided longitudinal technical quality-control information throughout the analytical period, allowing changes in instrument performance or analytical drift to be identified before acquisition of biological samples. In addition, three aliquots of a pooled urine quality-control (QC) sample were analyzed within each study-sample run: one at the beginning, one approximately halfway through the analytical sequence, and one at the end. These pooled QC samples were used to monitor spectral acquisition quality and instrument stability throughout the run and to verify that no relevant deterioration or analytical drift occurred during the measurement of the study samples.

Several different 1H NMR experiments were recorded in all the samples: a one-dimensional (1D) NOESY (Nuclear Overhauser Effect Spectroscopy) 32-scan NMR experiment was used to show the NMR spectrum quality (via the B.I. BioBankQC™ 1.0.0 Bruker, BioSpin GmbH, Rheinstetten, Germany) and to enable the metabolites quantification of the B.I. Quant-UR™ 1.1.0 (Bruker BioSpin GmbH, Rheinstetten, Germany). This 1D NOESY experiment was acquired using 32 scans, 4 dummy scans, a relaxation delay of 4.0 s, 64 k data points, a spectral width of 20 ppm, an acquisition time of 2.75 s, and a NOESY mixing time of 10 ms. These acquisition conditions form part of the standardized IVDr parameter set used in combination with the B.I. QUANT-UR quantification procedure. Moreover, a two-dimensional (2D) 2-scan JRES (J-RESolved spectroscopy), was included with the IVDr methods to analyze J-coupling constants. For samples with unknown peaks, we also included a 2D TOCSY (Total Correlation Spectroscopy) to identify the unknown metabolites.

B.I. QUANT-UR performed automated metabolite-specific spectral fitting using validated models developed for spectra acquired under standardized IVDr conditions. In addition to the calculated concentration, the software provided quality parameters for individual metabolite measurements. These included the signal correlation (ρ), which describes the agreement between the experimental metabolite signal and its calculated spectral fit, and the concentration error (Δ, mmol/L), which represented the concentration-equivalent difference between the experimental signal and the fitted signal.

At the individual-sample level, each 1D spectrum was evaluated using B.I. BioBankQC™ 1.0.0. The automated urine quality-control procedure evaluated compliance with the predefined NMR experimental parameters as well as several aspects of sample and spectral quality, including NMR experiment quality, sample-preparation quality, matrix identity, matrix integrity, potential contamination, protein background, and other parameters capable of affecting spectral reliability. The TSP reference signal additionally provided information regarding consistency of sample preparation. Absolute quantifications from 1H-NMR spectra were performed with Bruker IVDr software B.I. Quant-UR 1.1.0 to quantify urine metabolites (mmol/L units): Methylglutaconate, Phenyllactate, Aminobutyrate, Aminohippurate, Ethylphenol, Hydroxyhippurate, 4-Hydroxyphenylacetate, Hydroxyphenyllactate, Hydroxyphenylpyruvate, Pyridoxate, Aminolevulinate, Aminopentanoate, Acetate, Acetoacetate, Acetoine, Acetone, Adenine, Adenosine, Alanine, Allantoin, Arginine, Argininosuccinate, Benzoate, Betaine, Butyrate, Caffeine, Choline, Citraconate, Citrate, Citrulline, Creatine, Creatinine, Cystine, Cytosine, D-Galactonate, D-Galactose, D-Gluconate, D-Glucose, D-Lactose, D-Mandelate, D-Mannitol, D-Mannose, D-Panthenol, Dihydrothymine, Dihydrouracil, Dimethylamine, DL-Alloisoleucine, DL-Kynurenin, DL-Tyrosine, E-Glutaconate, Ethanol, Ethylmalonate, Formate, Fumarate, Galactitol, Glutamate, Glutamine, Glutarate, Glycerol, Glycine, Glycolate, Guanidinoacetate, Hippurate, Imidazole, Inosine, Isobutyrylglycine, Isopropanol, L-Ascorbate, L-Carnosine, L-Citramalate, L-Fucose, L-Homocystine, L-Isoleucine, L-Pyroglutamate, L-Threonate, L-Tryptophan, Lactate, Leucine, Maleate, Malate, Methanol, Methionine, Methylmalonate, Myo-Inositol, N-Acetylaspartate, N-Acetylglutamate, N-Acetylphenylalanine, N-Acetyltyrosine, N-Isovaleroylglycine, NN-Dimethylglycine, Neopterin, Orotate, Oxaloacetate, Pantothenate, Paracetamol, Paracetamol glucuronide, Phenylacetate, Phenylalanine, Phenylpyruvate, Pimelate, Proline betaine, Propionate, Propionylglycine, Propylene glycol, Pyrocatechol, Pyruvate, Quinolinate, Sarcosine, Succinate, Succinylacetone, Syringate, Tartarate, Taurine, Theobromine, Thymine, Thymol, Tiglylglycine, Trigonelline, Trimethylamine, Tyramine, Uracil, Uridine, Valine, and Xathurenate.

**Figure 7:**
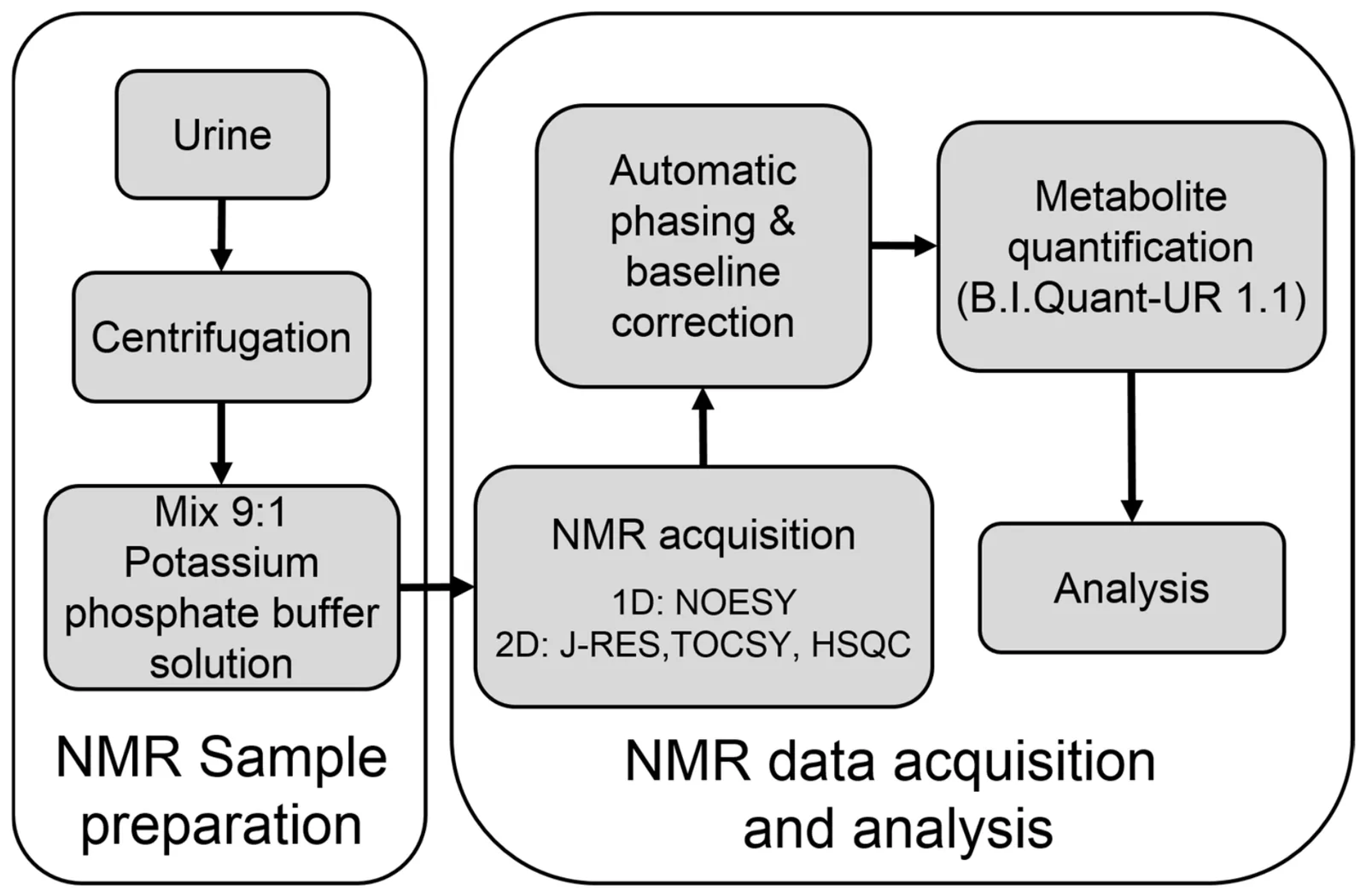
NMR analysis flowchart for human urine metabolomic studies.

A flowchart of the NMR analysis procedure is shown in Figure 7.

### 4.5. Statistical Analysis

NMR data were managed using SPSS version 28 (IBM Corp., Armonk, NY, USA), and the analysis was performed using Student’s *t*-test (STT) and Mann–Whitney test (MWT) to elucidate which metabolites could be relevant as hypertension biomarkers. Chi-squared test (CST) was used to compare the gender percentage between groups. One-way ANOVA test (OWAT), Kruskal–Wallis test (KWT), and Fisher’s exact test (FET) were used to analyze possible confounders regarding present comorbidities and drug treatments. *P*-values under 0.05 were considered statistically significant.

## Figures and Tables

**Table 1:** Characterization and comparison of sociodemographic, BP, biochemical and cognitive data between the NT vs. HTall groups, and the NT group vs. the HTo subgroup. Data are expressed as “mean ± S.E.” except for sex, expressed as “percent (*n*)”. Statistical analysis was performed using Student’s *t*-test (STT), Mann–Whitney test (MWT), or chi-squared test (CST). * *p* < 0.05 compared with NT.

PARAMETERS	NT	HTall	*p*-Value	HTo	*p*-Value
SOCIODEMOGRAPHIC DATA					
Number (*n*)	9	101	-	21	-
Age (years)	77.78 ± 3.03	84.08 ± 0.81	0.029 * (STT)	83.38 ± 2.52	0.207 (STT)
Sex, female (% (*n*))	44.40 (4)	71.30 (72)	0.132 (CST)	52.4 (11)	1.000 (CST)
Body Mass Index (kg/m^2^)	25.03 ± 1.88	27.48 ± 0.57	0.177 (STT)	26.19 ± 2.01	0.706 (STT)
BLOOD PRESSURE					
Systolic Blood Pressure (mmHg)	132.78 ± 6.03	122.81 ± 2	0.146 (STT)	119.48 ± 5.4	0.159 (STT)
Diastolic Blood Pressure (mmHg)	72.22 ± 3.9	69.38 ± 1.1	0.328 (MWT)	72.45 ± 2.33	0.660 (MWT)
BIOCHEMICAL TEST RESULTS					
Serum glucose (mg/dL)	96.46 ± 6.11	100.51 ± 2.68	0.972 (MWT)	95.89 ± 2.87	0.925 (STT)
Serum triglycerides (mg/dL)	116.06 ± 14.64	108.45 ± 4.84	0.532 (MWT)	99.67 ± 11.05	0.396 (STT)
Serum total cholesterol (mg/dL)	185.54 ± 8.57	156.97 ± 4.49	0.013 * (MWT)	186.46 ± 8.02	0.945 (STT)
Serum HDL cholesterol (mg/dL)	59.35 ± 4.49	53.31 ± 1.11	0.117 (STT)	57.7 ± 2.14	0.707 (STT)
Serum LDL cholesterol (mg/dL)	102.97 ± 7.29	81.96 ± 4.1	0.021 * (MWT)	108.83 ± 9.1	0.684 (STT)
COGNITIVE TESTS RESULTS					
Revised Addenbrooke’s Cognitive Test (ACE-R)	52.13 ± 5.63	47.7 ± 2.04	0.546 (STT)	42.1 ± 4.25	0.205 (STT)
Mini-Mental State Examination (MMSE)	20.89 ± 1.2	17.56 ± 0.61	0.028 * (STT)	15.14 ± 1.41	0.019 * (STT)
Global Deterioration Scale (GDS)	2.89 ± 0.31	3.02 ± 0.15	0.851 (MWT)	3.29 ± 0.34	0.533 (MWT)

**Table 2:** Characterization and comparison of pharmacological treatments between NT vs. HTall and NT vs. HTo individuals. Data are expressed as “percent (*n*)”. Statistical correlation analysis was performed using Fisher’s exact test (FET). * *p* < 0.05, ** *p* < 0.01, and *** *p* < 0.001 compared with NT. “-” means that the calculation is not applicable because *n* = 0 in one of the groups.

TREATMENTS	NT	HTall	*p*-Value NT vs. HTall	HTo	*p*-Value NT vs. HTo
Number (*n*)	9	101	-	21	
**Treatments for Hypertension/CVD**					
Antiarrhythmic drugs	0.0 (0)	3.0 (3)	1.000	0.0 (0)	-
Antianginal drugs	0.0 (0)	10.9 (11)	0.594	4.8 (1)	1.000
ACEi	0.0 (0)	25.7 (26)	0.112	19.0 (4)	0.287
ARA	0.0 (0)	48.5 (49)	0.004 **	47.6 (10)	0.013 *
BAA	0.0 (0)	25.7 (26)	0.112	4.8 (1)	1.000
CCB	0.0 (0)	29.7 (30)	0.110	9.5 (2)	1.000
DIU	0.0 (0)	70.3 (71)	<0.001 ***	47.6 (10)	0.013 *
Venotropics	22.2 (2)	15.8 (16)	0.639	14.3 (3)	0.622
AnCoa	11.1 (1)	56.4 (57)	0.012 *	38.1 (8)	0.210
**Treatments for metabolic diseases**					
Sulfonylureas	0.0 (0)	6.9 (7)	1.000	0.0 (0)	-
Biguanides	0.0 (0)	17.8 (18)	0.351	0.0 (0)	-
DPP-4 inhibitors	0.0 (0)	19.8 (20)	0.208	0.0 (0)	-
Insulin	0.0 (0)	6.9 (7)	1.000	0.0 (0)	-
Statins	22.2 (2)	49.5 (50)	0.167	0.0 (0)	0.083
**Treatments for respiratory diseases**					
Bronchodilators	0.0 (0)	13.9 (14)	0.600	0.0 (0)	-
**Treatments for central nervous system diseases**					
AChE inhibitors	0.0 (0)	5.0 (5)	1.000	9.5 (2)	1.000
MAO inhibitors	0.0 (0)	1.0 (1)	1.000	0.0 (0)	-
NMDA antagonist	0.0 (0)	6.9 (7)	1.000	4.8 (1)	1.000
Antiepileptics	11.1 (1)	10.9 (11)	1.000	14.3 (3)	1.000
Antipsychotics	33.3 (3)	22.8 (23)	0.439	42.9 (9)	0.704
Antidepressants	22.2 (2)	38.6 (39)	0.480	38.1 (8)	0.675

**Table 3:** Changes in urinary concentrations (mmol/L) of acetone, citrate, 4-hydroxyphenylacetate (4-HPAA), 3-methylglutaconate, N-isovaleroylglycine, oxaloacetate, phenylalanine, pyridoxate, and sarcosine urinary levels between HTall drug clusters: individuals treated with different drug combinations of ACEi, ARA, DIU, BAA, CCB and/or AnCoa vs. individuals not taking any of these drugs or drug combinations (“Without drugs”). The symbols indicate an increase (▲) or decrease (▼) in urinary metabolite concentrations vs. the “without drugs” subgroup. Data are expressed as mean ± s.e.m., followed by “percent of change” compared with unmedicated individuals. Statistical analysis was performed by STT. * *p* < 0.05; *** *p* < 0.001.

THERAPIES	Mean ± s.e.m.	Percent of Change	Mean ± s.e.m.	Percent of Change
	**Acetone**		**Citrate**	
Without drugs (5)	0.0120 ± 0.0024		1.9500 ± 0.6309	
ACEi (1)	0.0320 ± N.A.	▲ 166.7 *	1.7210 ± N.A.	▼ 11.7
ACEi+AnCoa (3)	0.0147 ± 0.0032	▲ 22.5	2.3460 ± 0.7316	▲ 20.3
ACEi+AnCoa+DIU (4)	0.0095 ± 0.0023	▼ 20.8	1.9490 ± 1.2700	▼ 0.1
ACEi+AnCoa+CCB+DIU (4)	0.0110 ± 0.0026	▼ 8.3	1.0310 ± 0.5524	▼ 47.1
ARA (5)	0.0148 ± 0.0027	▲ 23.3	1.6860 ± 0.5758	▼ 13.5
ARA+DIU (10)	0.0111 ± 0.0019	▼ 7.5	1.5303 ± 0.4317	▼ 21.5
ARA+AnCoa+DIU (9)	0.0124 ± 0.0019	▲ 3.3	1.5282 ± 0.5118	▼ 21.6
ARA+AnCoa+CCB (4)	0.0225 ± 0.0036	▲ 87.5 *	1.3235 ± 0.3295	▼ 32.1
ARA+AnCoa+DIU+CCB (4)	0.0108 ± 0.0029	▼ 10.0	0.9253 ± 0.4351	▼ 52.5
DIU (3)	0.0150 ± 0.0031	▲ 25.0	2.5773 ± 1.5544	▲ 32.2
DIU+AnCoa (5)	0.0134 ± 0.0022	▲ 11.7	0.7628 ± 0.3506	▼ 60.9
DIU+AnCoa+ARA (9)	0.0150 ± 0.0030	▲ 3.3	0.7735 ± 0.0265	▼ 21.6
DIU+AnCoa+BAA (2)	0.0120 ± 0.0024	▲ 25.0	1.9500 ± 0.6309	▼ 60.3
	**4-HPAA**		**3-Methylglutaconate**	
Without drugs (5)	0.0622 ± 0.0194		0.0800 ± 0.0385	
ACEi (1)	0.4630 ± N.A.	▲ 644.4 *	0.0740 ± N.A.	▼ 7.5
ACEi+AnCoa (3)	0.0387 ± 0.0195	▼ 37.8	0.0363 ± 0.0018	▼ 54.6
ACEi+AnCoa+DIU (4)	0.3098 ± 0.2839	▲ 398.1	0.0840 ± 0.0365	▲ 5.0
ACEi+AnCoa+CCB+DIU (4)	0.0733 ± 0.0441	▲ 17.8	0.0610 ± 0.0069	▼ 23.8
ARA (5)	0.0880 ± 0.0370	▲ 41.5	0.0600 ± 0.0283	▼ 25.0
ARA+DIU (10)	0.0668 ± 0.0156	▲ 7.4	0.0425 ± 0.0089	▼ 46.9
ARA+AnCoa+DIU (9)	0.1696 ± 0.1035	▲ 172.7	0.0567 ± 0.0078	▼ 29.1
ARA+AnCoa+CCB (4)	0.0263 ± 0.0263	▼ 57.7	0.1230 ± 0.0353	▲ 53.8
ARA+AnCoa+DIU+CCB (4)	0.1093 ± 0.0918	▲ 75.7	0.0350 ± 0.0120	▼ 56.3
DIU (3)	0.3550 ± 0.2478	▲ 470.7	0.0950 ± 0.0351	▲ 18.8
DIU+AnCoa (5)	0.0330 ± 0.0174	▼ 46.9	0.0476 ± 0.0190	▼ 40.5
DIU+AnCoa+ARA (9)	0.0975 ± 0.0185	▲ 172.7	0.0930 ± 0.0120	▼ 29.1
DIU+AnCoa+BAA (2)	0.0622 ± 0.0194	▲ 56.8	0.0800 ± 0.0385	▲ 16.3
	**N-Isovaleroylglycine**		**Oxaloacetate**	
Without drugs (5)	0.0056 ± 0.0056		0.4518 ± 0.1525	
ACEi (1)	0 ± N.A.	▼ 100.0	0.2030 ± N.A.	▼ 55.1
ACEi+AnCoa (3)	0.0027 ± 0.0027	▼ 51.8	0.1970 ± 0.0483	▼ 56.4
ACEi+AnCoa+DIU (4)	0.0033 ± 0.0033	▼ 41.1	0.0855 ± 0.0322	▼ 81.1
ACEi+AnCoa+CCB+DIU (4)	0.0165 ± 0.0079	▲ 194.6	0.2925 ± 0.1062	▼ 35.3
ARA (5)	0.0020 ± 0.0018	▼ 64.3	0.2294 ± 0.1058	▼ 49.2
ARA+DIU (10)	0.0031 ± 0.0021	▼ 44.6	0.2413 ± 0.0659	▼ 46.6
ARA+AnCoa+DIU (9)	0.0077 ± 0.0034	▲ 37.5	0.2578 ± 0.0903	▼ 42.9
ARA+AnCoa+CCB (4)	0.0095 ± 0.0055	▲ 69.6	0.7538 ± 0.2600	▲ 66.8
ARA+AnCoa+DIU+CCB (4)	0.0070 ± 0.0045	▲ 25.0	0.4378 ± 0.2212	▼ 3.1
DIU (3)	0.0180 ± 0.0080	▲ 221.4	0.5580 ± 0.2197	▲ 23.5
DIU+AnCoa (5)	0.0032 ± 0.0020	▼ 42.9	0.2286 ± 0.0700	▼ 49.4
DIU+AnCoa+ARA (9)	0.0070 ± 0.0070	▲ 37.5	0.5080 ± 0.2070	▼ 42.9
DIU+AnCoa+BAA (2)	0.0056 ± 0.0056	▲ 25.0	0.4518 ± 0.1525	▲ 12.4
	**Phenylalanine**		**Pyridoxate**	
Without drugs (5)	0.1258 ± 0.0681		0.0024 ± 0.0009	
ACEi (1)	0.2300 ± N.A.	▲ 82.8	0.0060 ± N.A.	▲ 150.0
ACEi+AnCoa (3)	0.0233 ± 0.0233	▼ 81.5	0.0060 ± 0.0026	▲ 150.0
ACEi+AnCoa+DIU (4)	0.1915 ± 0.0897	▲ 52.2	0.0108 ± 0.0074	▲ 350.0
ACEi+AnCoa+CCB+DIU (4)	0.0785 ± 0.0513	▼ 37.6	0.0100 ± 0.0071	▲ 316.7
ARA (5)	0.0864 ± 0.0204	▼ 31.3	0.0048 ± 0.0038	▲ 100.0
ARA+DIU (10)	0.1518 ± 0.0672	▲ 20.7	0.0129 ± 0.0034	▲ 437.5 *
ARA+AnCoa+DIU (9)	0.0640 ± 0.0274	▼ 49.1	0.0057 ± 0.0018	▲ 137.5
ARA+AnCoa+CCB (4)	0.2013 ± 0.0818	▲ 60.0	0.0118 ± 0.0015	▲ 391.7 ***
ARA+AnCoa+DIU+CCB (4)	0.0423 ± 0.0222	▼ 66.4	0.0043 ± 0.0020	▲ 79.2
DIU (3)	0.0660 ± 0.0333	▼ 47.5	0.0083 ± 0.0027	▲ 245.8 *
DIU+AnCoa (5)	0.0576 ± 0.0251	▼ 54.2	0.0076 ± 0.0039	▲ 216.7
DIU+AnCoa+ARA (9)	0.0720 ± 0.0720	▼ 49.1	0.0015 ± 0.0015	▲ 137.5
DIU+AnCoa+BAA (2)	0.1258 ± 0.0681	▼ 42.8	0.0024 ± 0.0009	▼ 37.5
	**Sarcosine**			
Without drugs (5)	0.0110 ± 0.0060			
ACEi (1)	0.0080 ± N.A.	▼ 27.3		
ACEi+AnCoa (3)	0.0047 ± 0.0009	▼ 57.3		
ACEi+AnCoa+DIU (4)	0.0155 ± 0.0096	▲ 40.9		
ACEi+AnCoa+CCB+DIU (4)	0.0385 ± 0.0301	▲ 250.0		
ARA (5)	0.0082 ± 0.0029	▼ 25.5		
ARA+DIU (10)	0.0079 ± 0.0020	▼ 28.2		
ARA+AnCoa+DIU (9)	0.0106 ± 0.0032	▼ 3.6		
ARA+AnCoa+CCB (4)	0.0175 ± 0.0086	▲ 59.1		
ARA+AnCoa+DIU+CCB (4)	0.0068 ± 0.0021	▼ 38.2		
DIU (3)	0.0080 ± 0.0032	▼ 27.3		
DIU+AnCoa (5)	0.0050 ± 0.0016	▼ 54.5		
DIU+AnCoa+ARA (9)	0.0275 ± 0.0155	▼ 3.6		
DIU+AnCoa+BAA (2)	0.0110 ± 0.0060	▲ 150.0		

## Data Availability

The data that support the findings of this study are available on request from the corresponding author, I. Verde. The data are not publicly available due to restrictions of data protection law (Portugal) because they contain information that could compromise the privacy of research participants.

## Supplementary Materials

The following supporting information can be downloaded at: https://www.mdpi.com/article/10.3390/molecules31183349/s1. Table S1. Concentrations of urinary metabolites (mmol/L) and comparison between NT (*n* = 9) and HTo (*n* = 21) groups. Data are expressed as “mean ± s.e.m”. Statistical analysis between groups was performed using Student’s *t*-test (STT) or Mann-Whitney Test (MWT). Table S2. Concentrations of urinary metabolites (mmol/L) and comparison between NT (*n* = 9) and HTall (*n* = 101) groups. Data are expressed as “mean ± s.e.m”. Statistical analysis between groups was performed using Student’s *t*-test (STT) or Mann-Whitney Test (MWT). Table S3. Urinary concentration levels (mmol/L) of metabolites in HTo subgroup and subgroups of hypertensive patients with dyslipidemia (with or without other comorbidities): hypertensive individuals with dyslipidemia only (HTdl), hypertensive individuals with dyslipidemia and diabetes (HTdb•dl) and hypertensive individuals with dyslipidemia and one cardiac disease (HTcd•dl). Data are expressed as “mean ± s.e.m”. Statistical analysis was performed using Kruskal-Wallis test (KWT), with P value indicated between parenthesis after each metabolite name, followed by post hoc Dunn-Bonferroni Test. * *p* < 0.05 compared with HTo; # *p* < 0.05 compared with HTdl; & *p* < 0.05 compared with HTdb•dl. Table S4. Urinary concentration levels (mmol/L) of metabolites in HTo subgroup and subgroups of hypertensive patients with diabetes (with or without other comorbidities): hypertensive individuals with diabetes only (HTdb), hypertensive individuals with diabetes and dyslipidemia (HTdb•dl) and hypertensive individuals with diabetes and one cardiac disease (HTcd•db). Data are expressed as “mean ± s.e.m”. Statistical analysis was performed using Kruskal-Wallis test (KWT), with P value indicated between parenthesis after each metabolite name, followed by post hoc Dunn-Bonferroni Test. * *p* < 0.05 compared with HTo; # *p* < 0.05 compared with HTdb. Table S5. Urinary concentration levels (mmol/L) of metabolites in HTo subgroup and subgroups of hypertensive patients with one cardiac disease (with or without other comorbidities): hypertensive individuals with one cardiac disease only (HTcd), hypertensive individuals with one cardiac disease and dyslipidemia (HTcd•dl) and hypertensive individuals with one cardiac disease and diabetes (HTcd•db). Data are expressed as “mean ± s.e.m”. Statistical analysis was performed using Kruskal-Wallis test (KWT), with *p* value indicated between parenthesis after each metabolite name, followed by post hoc Dunn-Bonferroni Test. * *p* < 0.05 compared with HTo; # *p* < 0.05 compared with HTcd; & *p* < 0.05 compared with HTcd•dl. Table S6. Receiver operating characteristic (ROC) analysis of acetone, citrate, 4-HPAA, methylglutaconate, N-isovaleroylglycine, oxaloacetate, phenylalanine, pyridoxate, and sarcosine between NT and HTall participants. Table S7. Receiver operating characteristic (ROC) analysis of the metabolites that showed significant differences between NT and HTo participants. Figure S1. Representative 600 MHz 1H-NMR NOESY spectrum of a urine sample. Numbers indicate the following metabolites: (1) Acetate; (2) Acetoacetate; (3) Acetone; (4) Alanine; (5) Allantoin; (6) Citrate; (7) Creatine; (8) Creatinine; (9) Dimethylamine; (10) Ethanol; (11) Formate; (12) Glycine; (13) Hippurate; (14) 4-Hydroxyphenylacetate; (15) Isoleucine; (16) Lactate; (17) Phenylalanine; (18) Phenyl lactate; (19) Pyruvate; (20) Succinate; (21) Taurine; (22) Valine; (23) Sugars (glucose, galactose, mannitol).

## Abbreviation List

1D	One-Dimensional
2D	Two-Dimensional
4-HPAA	4-Hydroxyphenylacetate
Ace	Acetone
ACEi	Angiotensin-Converting Enzyme inhibitors
ACE-R	Revised Addenbrooke’s Cognitive Test
AChE	Acetylcholinesterase
AnCoa	Anticoagulants
ARA	Angiotensin II Receptor Antagonists
AUC	Area Under the Curve
AUROC	Area Under the Receiver Operating Characteristic Curve
BAA	Beta-Adrenergic Antagonists
BMI	Body Mass Index
BP	Blood Pressure
CCB	Calcium Channel Blockers
Cit	Citrate
CST	Chi-Squared Test
CVMD	Cardiovascular or Metabolic Diseases
DBP	Diastolic Blood Pressure
DIU	Diuretics
DPP-4	Dipeptidyl peptidase-4
EBIcohort	Elders from Beira Interior cohort
FET	Fisher’s Exact Test
GDS	Global Deterioration Scale
HDL cholesterol	High-Density Lipoprotein cholesterol
HT	Hypertension
HTall	Group consisting of participants diagnosed with hypertension (with and without comorbidities)
HTo	Subgroup (inside HTall group) of participants diagnosed with hypertension, without any cardiovascular or metabolic comorbidities.
HTdl	Subgroup of participants diagnosed with hypertension + dyslipidemia, without other cardiovascular or metabolic comorbidities (namely, diabetes or cardiovascular diseases)
HTdb	Subgroup of participants diagnosed with hypertension + diabetes, without other cardiovascular or metabolic comorbidities (namely, dyslipidemia or cardiovascular diseases)
HTcd	Subgroup of participants diagnosed with hypertension + one cardiovascular disease (heart failure, arrhythmia, or history of acute myocardial infarction), without other cardiovascular or metabolic comorbidities (namely dyslipidemia or diabetes)
HTdb•dl	Subgroup of participants diagnosed with hypertension + dyslipidemia + diabetes, without other cardiovascular or metabolic comorbidities (namely, cardiovascular diseases)
HTcd•dl	Subgroup of participants diagnosed with hypertension + dyslipidemia + one cardiovascular disease (heart failure, arrhythmia, or history of acute myocardial infarction), without other cardiovascular or metabolic comorbidities (namely, diabetes)
HTcd•db	Subgroup of participants diagnosed with hypertension + diabetes + one cardiovascular disease (heart failure, arrhythmia, or history of acute myocardial infarction), without other cardiovascular or metabolic comorbidities (namely, dyslipidemia)
JRES	J-RESolved spectroscopy
KWT	Kruskal–Wallis test
LDL cholesterol	Low-Density Lipoprotein cholesterol
LTCF	Long-Term Care Facilities
MAO	Monoamine oxidase
MG	Methylglutaconate
MMSE	Mini-Mental State Examination
MWT	Mann–Whitney Test
N-IVG	N-Isovaleroylglycine
NMDA	N-Methyl-D-Aspartate
NMR	Nuclear Magnetic Resonance
NOESY	Nuclear Overhauser Effect Spectroscopy
NT	Normotensive
OWAT	One-way ANOVA test
Oxa	Oxaloacetate
Phe	Phenylalanine
Pyr	Pyridoxate
S.E.	Standard Error
Sar	Sarcosine
SBP	Systolic Blood Pressure
STT	Student *t*-test
TOCSY	Total Correlation Spectroscopy
WHO	World Health Organization
