# Supplementary material for: Searching for Metabolic Markers for Hypertension in Human Urine
Source: Molecules. 2026 Sep 21;31(18):3349. doi: 10.3390/molecules31183349 (PMC13610069; doi:10.3390/molecules31183349)
Supplement: Supplementary file 1 [file molecules-31-03349-s001.zip › 2026set11 Supplementary Figures.pdf]

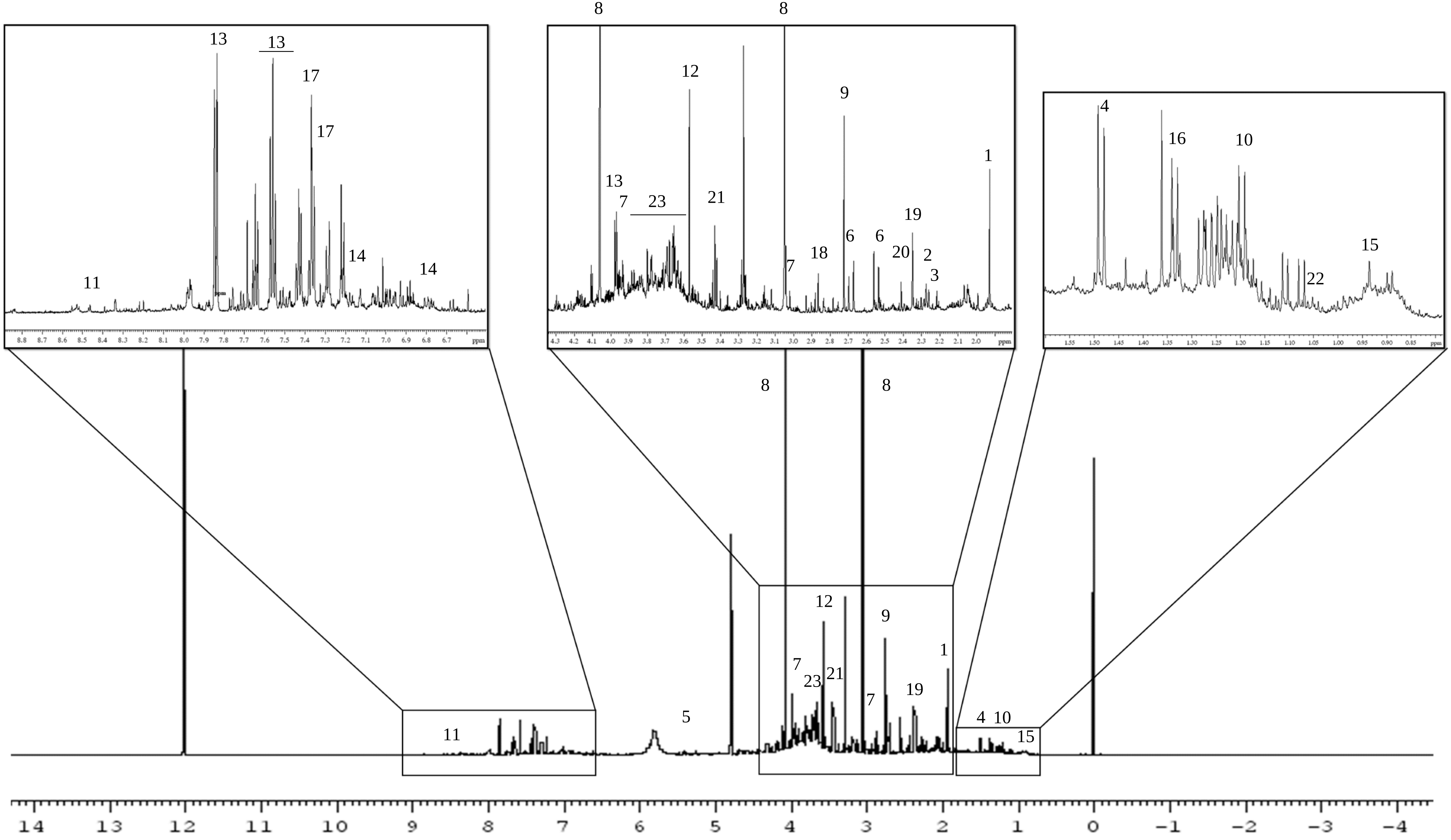
13
13
17
17
14
14
11
8
8
12
9
1
13
7
23
21
19
6
6
20
2
3
7
18
4
16
10
15
22
8
8
12
9
1
7
23
21
19
7
5
11
4
10
15
14 13 12 11 10 9 8 7 6 5 4 3 2 1 0 -1 -2 -3 -4

Figure S1. Representative 600 MHz ^{1}H-NMR NOESY spectrum of a urine sample. Numbers indicate the following metabolites:

1) Acetate; 2) Acetoacetate; 3) Acetone; 4) Alanine; 5) Allantoin; 6) Citrate; 7) Creatine; 8) Creatinine; 9) Dimethyamine; 10) Ethanol; 11) Formate; 12) Glycine; 13) Hippurate; 14) 4-Hydroxyphenylacetate; 15) Isoleucine; 16) Lactate; 17) Phenylalanine; 18) Phenyllactate; 19) Pyruvate; 20) Succinate; 21) Taurine; 22) Valine; 23) Sugars (glucose, galactose, manitol)
