# Supplementary material for: Searching for Metabolic Markers for Hypertension in Human Urine
Source: Molecules. 2026 Sep 21;31(18):3349. doi: 10.3390/molecules31183349 (PMC13610069; doi:10.3390/molecules31183349)
Supplement: Supplementary file 1 [file molecules-31-03349-s001.zip › 2026set11 Supplementary Tables.pdf]

Table S1. Concentrations of urinary metabolites (mmol/L) and comparison between NT (n = 9) and HTo (n = 21) groups. Data are expressed as "mean ± s.e.m". Statistical analysis between groups was performed using Student's t-test (STT) or Mann-Whitney Test (MWT).

Metabolite	NT	HTo	P-value	Test
Methylglutaconate	0.097 ± 0.020	0.050 ± 0.005	0.014	MWT
Phenyllactate	0.058 ± 0.033	0.021 ± 0.009	0.722	MWT
Aminobutyrate	0.019 ± 0.019	0.019 ± 0.012	0.929	MWT
Aminohippurate	0.000 ± 0.000	0.000 ± 0.000	1.000	MWT
Ethylphenol	0.000 ± 0.000	0.000 ± 0.000	1.000	MWT
Hydroxyhippurate	0.111 ± 0.069	0.056 ± 0.026	0.790	MWT
Hydroxyphenylacetate	0.310 ± 0.157	0.133 ± 0.049	0.045	MWT
Hydroxyphenyllactate	0.000 ± 0.000	0.007 ± 0.007	0.859	MWT
Hydroxyphenylpyruvate	0.016 ± 0.011	0.002 ± 0.002	0.449	MWT
Pyridoxate	0.027 ± 0.012	0.007 ± 0.002	0.045	MWT
Aminolevulinate	0.000 ± 0.000	0.000 ± 0.000	0.689	MWT
Aminopentanoate	0.000 ± 0.000	0.000 ± 0.000	1.000	MWT
Acetate	0.102 ± 0.019	0.365 ± 0.113	0.476	MWT
Acetoacetate	0.149 ± 0.029	0.115 ± 0.015	0.260	STT
Acetoine	0.000 ± 0.000	0.000 ± 0.000	1.000	MWT
Acetone	0.030 ± 0.008	0.013 ± 0.002	0.018	MWT
Adenine	0.005 ± 0.001	0.005 ± 0.001	0.929	MWT
Adenosine	0.000 ± 0.000	0.000 ± 0.000	1.000	MWT
Alanine	0.337 ± 0.100	0.258 ± 0.044	0.476	MWT
Allantoin	0.069 ± 0.016	0.094 ± 0.015	0.263	MWT
Arginine	0.460 ± 0.084	0.417 ± 0.044	0.621	STT
Argininosuccinate	0.000 ± 0.000	0.002 ± 0.002	0.859	MWT
Benzoate	0.003 ± 0.003	0.034 ± 0.019	0.422	MWT
Betaine	0.142 ± 0.045	0.108 ± 0.041	0.372	MWT
Butyrate	0.009 ± 0.006	0.025 ± 0.008	0.449	MWT
Caffeine	0.264 ± 0.113	0.125 ± 0.018	0.349	MWT
Choline	0.000 ± 0.000	0.024 ± 0.008	0.070	MWT
Citraconate	0.000 ± 0.000	0.000 ± 0.000	1.000	MWT
Citrate	3.653 ± 1.159	1.355 ± 0.217	0.036	MWT
Citrulline	0.389 ± 0.123	0.220 ± 0.033	0.218	STT
Creatine	0.228 ± 0.134	0.261 ± 0.114	0.824	MWT
Creatinine	8.034 ± 1.823	5.458 ± 0.559	0.304	MWT
Cystine	0.000 ± 0.000	0.000 ± 0.000	1.000	MWT
Cytosine	0.004 ± 0.004	0.002 ± 0.001	0.563	MWT
D-Galactonate	0.010 ± 0.010	0.012 ± 0.010	0.965	MWT
D-Galactose	0.014 ± 0.007	0.011 ± 0.006	0.397	MWT
D-Gluconate	0.047 ± 0.032	0.024 ± 0.014	0.824	MWT
D-Glucose	0.349 ± 0.088	0.273 ± 0.038	0.563	MWT

D-Lactose	0.143 ± 0.041	0.131 ± 0.026	0.965	MWT
D-Mandelate	0.000 ± 0.000	0.000 ± 0.000	0.859	MWT
D-Mannitol	0.080 ± 0.080	0.150 ± 0.109	1.000	MWT
D-Mannose	0.007 ± 0.004	0.011 ± 0.006	0.689	MWT
D-Panthenol	0.000 ± 0.000	0.000 ± 0.000	1.000	MWT
Dihydrothymine	0.439 ± 0.376	0.083 ± 0.018	0.929	MWT
Dihydrouracil	0.007 ± 0.007	0.004 ± 0.002	0.965	MWT
Dimethylamine	0.409 ± 0.110	0.300 ± 0.031	0.859	MWT
DL-Alloisoleucine	0.016 ± 0.016	0.003 ± 0.002	0.965	MWT
DL-Kynurenin	0.000 ± 0.000	0.000 ± 0.000	1.000	MWT
DL-Tyrosine	0.000 ± 0.000	0.000 ± 0.000	1.000	MWT
E-Glutaconate	0.000 ± 0.000	0.000 ± 0.000	1.000	MWT
Ethanol	0.000 ± 0.000	0.004 ± 0.004	0.859	MWT
Ethylmalonate	0.050 ± 0.011	0.031 ± 0.006	0.125	MWT
Formate	0.168 ± 0.040	0.298 ± 0.078	0.533	MWT
Fumarate	0.007 ± 0.003	0.005 ± 0.001	0.859	MWT
Galactitol	0.000 ± 0.000	0.000 ± 0.000	1.000	MWT
Glutamate	0.000 ± 0.000	0.012 ± 0.012	0.859	MWT
Glutamine	0.000 ± 0.000	0.063 ± 0.063	0.859	MWT
Glutarate	0.061 ± 0.023	0.031 ± 0.006	0.304	MWT
Glycerol	0.000 ± 0.000	0.000 ± 0.000	1.000	MWT
Glycine	0.807 ± 0.251	0.628 ± 0.084	0.965	MWT
Glycolate	0.724 ± 0.198	0.507 ± 0.112	0.226	MWT
Guanidinoacetate	0.510 ± 0.168	0.321 ± 0.046	0.263	MWT
Hippurate	4.182 ± 2.127	1.440 ± 0.339	0.349	MWT
Imidazole	0.091 ± 0.053	0.037 ± 0.011	0.193	MWT
Inosine	0.025 ± 0.008	0.021 ± 0.004	0.594	MWT
Isobutyrylglycine	0.000 ± 0.000	0.000 ± 0.000	1.000	MWT
Isopropanol	0.000 ± 0.000	0.000 ± 0.000	1.000	MWT
L-Ascorbate	0.000 ± 0.000	0.000 ± 0.000	1.000	MWT
L-Carnosine	0.000 ± 0.000	0.000 ± 0.000	1.000	MWT
L-Citramalate	0.181 ± 0.049	0.144 ± 0.012	0.859	MWT
L-Fucose	0.382 ± 0.079	0.371 ± 0.076	0.932	STT
L-Homocystine	0.000 ± 0.000	0.000 ± 0.000	1.000	MWT
L-Isoleucine	0.034 ± 0.023	0.009 ± 0.002	0.533	MWT
L-Pyroglutamate	0.000 ± 0.000	0.005 ± 0.004	0.689	MWT
L-Threonate	0.000 ± 0.000	0.000 ± 0.000	1.000	MWT
L-Tryptophan	0.073 ± 0.036	0.043 ± 0.013	0.625	MWT
Lactate	0.168 ± 0.040	0.193 ± 0.039	0.929	MWT
Leucine	0.046 ± 0.016	0.033 ± 0.004	0.756	MWT
Maleate	0.004 ± 0.001	0.005 ± 0.000	0.164	MWT
Malate	0.000 ± 0.000	0.000 ± 0.000	1.000	MWT

Methanol	0.187 ± 0.060	0.198 ± 0.062	0.965	MWT
Methionine	0.019 ± 0.011	0.002 ± 0.002	0.283	MWT
Methylmalonate	0.064 ± 0.035	0.061 ± 0.014	0.563	MWT
Myo-Inositol	0.000 ± 0.000	0.050 ± 0.041	0.689	MWT
N-Acetylaspartate	0.065 ± 0.016	0.046 ± 0.004	0.304	MWT
N-Acetylglutamate	0.000 ± 0.000	0.002 ± 0.002	0.859	MWT
N-Acetylphenylalanine	0.000 ± 0.000	0.005 ± 0.005	0.859	MWT
N-Acetyltyrosine	0.000 ± 0.000	0.000 ± 0.000	1.000	MWT
N-Isovaleroylglycine	0.023 ± 0.008	0.007 ± 0.003	0.032	MWT
NN-Dimethylglycine	0.062 ± 0.014	0.049 ± 0.005	0.594	MWT
Neopterin	0.154 ± 0.106	0.036 ± 0.016	0.824	MWT
Orotate	0.003 ± 0.000	0.003 ± 0.000	0.533	MWT
Oxaloacetate	0.584 ± 0.119	0.282 ± 0.046	<.001	MWT
Pantothenate	0.067 ± 0.018	0.047 ± 0.004	0.756	MWT
Paracetamol	0.000 ± 0.000	0.011 ± 0.011	0.563	MWT
Paracetamol glucuronide	0.000 ± 0.000	0.004 ± 0.004	0.859	MWT
Phenylacetate	0.035 ± 0.023	0.009 ± 0.003	0.244	MWT
Phenylalanine	0.208 ± 0.091	0.089 ± 0.033	0.028	MWT
Phenylpyruvate	0.047 ± 0.017	0.038 ± 0.009	0.563	MWT
Pimelate	0.000 ± 0.000	0.000 ± 0.000	1.000	MWT
Proline Betaine	0.160 ± 0.050	0.161 ± 0.046	0.824	MWT
Propionate	0.019 ± 0.008	0.014 ± 0.003	0.965	MWT
Propionylglycine	0.000 ± 0.000	0.000 ± 0.000	1.000	MWT
Propylene Glycol	0.041 ± 0.015	0.047 ± 0.011	1.000	MWT
Pyrocatechol	0.000 ± 0.000	0.000 ± 0.000	1.000	MWT
Pyruvate	0.050 ± 0.013	0.026 ± 0.003	0.050	MWT
Quinolinate	0.000 ± 0.000	0.000 ± 0.000	1.000	MWT
Sarcosine	0.004 ± 0.001	0.010 ± 0.002	0.019	MWT
Succinate	0.097 ± 0.016	0.097 ± 0.017	0.449	MWT
Succinylacetone	0.000 ± 0.000	0.002 ± 0.002	0.859	MWT
Syringate	0.142 ± 0.072	0.094 ± 0.020	0.824	MWT
Tartarate	0.077 ± 0.066	0.039 ± 0.019	0.965	MWT
Taurine	0.545 ± 0.132	0.699 ± 0.116	0.594	MWT
Theobromine	0.084 ± 0.017	0.066 ± 0.012	0.164	MWT
Thymine	0.005 ± 0.003	0.002 ± 0.001	0.263	MWT
Thymol	0.121 ± 0.058	0.060 ± 0.012	0.226	MWT
Tiglylglycine	0.003 ± 0.003	0.002 ± 0.002	0.625	MWT
Trigonelline	0.248 ± 0.112	0.117 ± 0.025	0.226	MWT
Trimethylamine	0.009 ± 0.003	0.025 ± 0.013	0.790	MWT
Tyramine	0.000 ± 0.000	0.005 ± 0.005	0.859	MWT
Uracil	0.345 ± 0.180	0.179 ± 0.052	0.657	MWT
Uridine	0.027 ± 0.007	0.024 ± 0.006	0.594	MWT

Valine	0.028 ± 0.005	0.020 ± 0.003	0.137	STT
Xanthurenate	0.040 ± 0.009	0.023 ± 0.003	0.114	MWT

Table S2. Concentrations of urinary metabolites (mmol/L) and comparison between NT (n = 9) and HTall (n = 101) groups. Data are expressed as "mean ± s.e.m". Statistical analysis between groups was performed using Student's t-test (STT) or Mann-Whitney Test (MWT).

Metabolite	NT	HTall	P-value	Test
Methylglutaconate	0.097 ± 0.020	0.055 ± 0.004	0.008	MWT
Phenyllactate	0.058 ± 0.033	0.019 ± 0.003	0.668	MWT
Aminobutyrate	0.019 ± 0.019	0.011 ± 0.003	0.962	MWT
Aminohippurate	0.000 ± 0.000	0.000 ± 0.000	1.000	MWT
Ethylphenol	0.000 ± 0.000	0.000 ± 0.000	1.000	MWT
Hydroxyhippurate	0.111 ± 0.069	0.057 ± 0.018	0.513	MWT
Hydroxyphenylacetate	0.310 ± 0.157	0.112 ± 0.020	0.009	MWT
Hydroxyphenyllactate	0.000 ± 0.000	0.003 ± 0.002	0.545	MWT
Hydroxyphenylpyruvate	0.016 ± 0.011	0.004 ± 0.001	0.197	MWT
Pyridoxate	0.027 ± 0.012	0.009 ± 0.001	0.057	MWT
Aminolevulinate	0.000 ± 0.000	0.001 ± 0.000	0.383	MWT
Aminopentanoate	0.000 ± 0.000	0.000 ± 0.000	1.000	MWT
Acetate	0.102 ± 0.019	0.404 ± 0.079	0.593	MWT
Acetoacetate	0.149 ± 0.029	0.111 ± 0.009	0.075	MWT
Acetoine	0.000 ± 0.000	0.000 ± 0.000	1.000	MWT
Acetone	0.030 ± 0.008	0.013 ± 0.001	0.004	MWT
Adenine	0.005 ± 0.001	0.010 ± 0.003	0.784	MWT
Adenosine	0.000 ± 0.000	0.000 ± 0.000	1.000	MWT
Alanine	0.337 ± 0.100	0.209 ± 0.017	0.152	MWT
Allantoin	0.069 ± 0.016	0.090 ± 0.006	0.261	MWT
Arginine	0.460 ± 0.084	0.367 ± 0.024	0.150	MWT
Argininosuccinate	0.000 ± 0.000	0.001 ± 0.001	0.671	MWT
Benzoate	0.003 ± 0.003	0.047 ± 0.015	0.204	MWT
Betaine	0.142 ± 0.045	0.113 ± 0.019	0.167	MWT
Butyrate	0.009 ± 0.006	0.019 ± 0.003	0.448	MWT
Caffeine	0.264 ± 0.113	0.139 ± 0.012	0.310	MWT
Choline	0.000 ± 0.000	0.020 ± 0.003	0.023	MWT
Citraconate	0.000 ± 0.000	0.000 ± 0.000	1.000	MWT
Citrate	3.653 ± 1.159	1.396 ± 0.129	0.015	MWT
Citrulline	0.389 ± 0.123	0.212 ± 0.017	0.261	MWT
Creatine	0.228 ± 0.134	0.320 ± 0.058	0.574	MWT
Creatinine	8.034 ± 1.823	5.565 ± 0.364	0.125	MWT
Cystine	0.000 ± 0.000	0.002 ± 0.001	0.671	MWT
Cytosine	0.004 ± 0.004	0.004 ± 0.001	0.389	MWT
D-Galactonate	0.010 ± 0.010	0.009 ± 0.004	0.739	MWT

D-Galactose	0.014 ± 0.007	0.009 ± 0.002	0.187	MWT
D-Gluconate	0.047 ± 0.032	0.061 ± 0.014	0.825	MWT
D-Glucose	0.349 ± 0.088	0.428 ± 0.142	0.398	MWT
D-Lactose	0.143 ± 0.041	0.103 ± 0.010	0.220	MWT
D-Mandelate	0.000 ± 0.000	0.001 ± 0.000	0.383	MWT
D-Mannitol	0.080 ± 0.080	0.283 ± 0.080	0.509	MWT
D-Mannose	0.007 ± 0.004	0.010 ± 0.003	0.454	MWT
D-Panthenol	0.000 ± 0.000	0.001 ± 0.001	0.765	MWT
Dihydrothymine	0.439 ± 0.376	0.106 ± 0.042	0.647	MWT
Dihydrouracil	0.007 ± 0.007	0.003 ± 0.001	0.896	MWT
Dimethylamine	0.409 ± 0.110	0.281 ± 0.016	0.582	MWT
DL-Alloisoleucine	0.016 ± 0.016	0.005 ± 0.001	0.866	MWT
DL-Kynurenin	0.000 ± 0.000	0.000 ± 0.000	1.000	MWT
DL-Tyrosine	0.000 ± 0.000	0.000 ± 0.000	1.000	MWT
E-Glutaconate	0.000 ± 0.000	0.000 ± 0.000	1.000	MWT
Ethanol	0.000 ± 0.000	0.001 ± 0.001	0.765	MWT
Ethylmalonate	0.050 ± 0.011	0.025 ± 0.003	0.007	MWT
Formate	0.168 ± 0.040	0.163 ± 0.026	0.182	MWT
Fumarate	0.007 ± 0.003	0.005 ± 0.001	0.583	MWT
Galactitol	0.000 ± 0.000	0.002 ± 0.002	0.602	MWT
Glutamate	0.000 ± 0.000	0.002 ± 0.002	0.765	MWT
Glutamine	0.000 ± 0.000	0.038 ± 0.022	0.602	MWT
Glutarate	0.061 ± 0.023	0.028 ± 0.003	0.130	MWT
Glycerol	0.000 ± 0.000	0.011 ± 0.010	0.671	MWT
Glycine	0.807 ± 0.251	0.448 ± 0.037	0.245	MWT
Glycolate	0.724 ± 0.198	0.381 ± 0.030	0.034	MWT
Guanidinoacetate	0.510 ± 0.168	0.284 ± 0.019	0.048	MWT
Hippurate	4.182 ± 2.127	1.207 ± 0.127	0.103	MWT
Imidazole	0.091 ± 0.053	0.038 ± 0.005	0.262	MWT
Inosine	0.025 ± 0.008	0.028 ± 0.003	0.887	MWT
Isobutyrylglycine	0.000 ± 0.000	0.000 ± 0.000	1.000	MWT
Isopropanol	0.000 ± 0.000	0.003 ± 0.002	0.602	MWT
L-Ascorbate	0.000 ± 0.000	0.000 ± 0.000	1.000	MWT
L-Carnosine	0.000 ± 0.000	0.000 ± 0.000	1.000	MWT
L-Citramalate	0.181 ± 0.049	0.137 ± 0.010	0.392	MWT
L-Fucose	0.382 ± 0.079	0.331 ± 0.024	0.266	MWT
L-Homocystine	0.000 ± 0.000	0.000 ± 0.000	1.000	MWT
L-Isoleucine	0.034 ± 0.023	0.008 ± 0.001	0.262	MWT
L-Pyroglutamate	0.000 ± 0.000	0.012 ± 0.005	0.417	MWT
L-Threonate	0.000 ± 0.000	0.025 ± 0.012	0.497	MWT
L-Tryptophan	0.073 ± 0.036	0.041 ± 0.004	0.603	MWT
Lactate	0.168 ± 0.040	0.209 ± 0.027	0.639	MWT

Leucine	0.046 ± 0.016	0.029 ± 0.002	0.287	MWT
Maleate	0.004 ± 0.001	0.008 ± 0.001	0.172	MWT
Malate	0.000 ± 0.000	0.000 ± 0.000	1.000	MWT
Methanol	0.187 ± 0.060	0.148 ± 0.017	0.545	MWT
Methionine	0.019 ± 0.011	0.001 ± 0.001	0.003	MWT
Methylmalonate	0.064 ± 0.035	0.046 ± 0.006	0.991	MWT
Myo-Inositol	0.000 ± 0.000	0.010 ± 0.009	0.671	MWT
N-Acetylaspartate	0.065 ± 0.016	0.045 ± 0.003	0.163	MWT
N-Acetylglutamate	0.000 ± 0.000	0.001 ± 0.001	0.497	MWT
N-Acetylphenylalanine	0.000 ± 0.000	0.010 ± 0.004	0.325	MWT
N-Acetyltyrosine	0.000 ± 0.000	0.000 ± 0.000	1.000	MWT
N-Isovaleroylglycine	0.023 ± 0.008	0.007 ± 0.001	0.008	MWT
NN-Dimethylglycine	0.062 ± 0.014	0.048 ± 0.003	0.326	MWT
Neopterin	0.154 ± 0.106	0.031 ± 0.012	0.969	MWT
Orotate	0.003 ± 0.000	0.010 ± 0.002	0.782	MWT
Oxaloacetate	0.584 ± 0.119	0.306 ± 0.026	0.001	MWT
Pantothenate	0.067 ± 0.018	0.044 ± 0.002	0.287	MWT
Paracetamol	0.000 ± 0.000	0.007 ± 0.003	0.217	MWT
Paracetamol glucuronide	0.000 ± 0.000	0.056 ± 0.039	0.325	MWT
Phenylacetate	0.035 ± 0.023	0.022 ± 0.005	0.965	MWT
Phenylalanine	0.208 ± 0.091	0.085 ± 0.011	0.020	MWT
Phenylpyruvate	0.047 ± 0.017	0.037 ± 0.004	0.505	MWT
Pimelate	0.000 ± 0.000	0.000 ± 0.000	1.000	MWT
Proline Betaine	0.160 ± 0.050	0.181 ± 0.024	0.917	MWT
Propionate	0.019 ± 0.008	0.014 ± 0.001	0.929	MWT
Propionylglycine	0.000 ± 0.000	0.000 ± 0.000	1.000	MWT
Propylene Glycol	0.041 ± 0.015	0.053 ± 0.011	0.356	MWT
Pyrocatechol	0.000 ± 0.000	0.000 ± 0.000	1.000	MWT
Pyruvate	0.050 ± 0.013	0.026 ± 0.002	0.034	MWT
Quinolinate	0.000 ± 0.000	0.003 ± 0.002	0.671	MWT
Sarcosine	0.004 ± 0.001	0.010 ± 0.002	0.066	MWT
Succinate	0.097 ± 0.016	0.096 ± 0.009	0.218	MWT
Succinylacetone	0.000 ± 0.000	0.024 ± 0.005	0.103	MWT
Syringate	0.142 ± 0.072	0.090 ± 0.010	0.798	MWT
Tartarate	0.077 ± 0.066	0.038 ± 0.009	0.552	MWT
Taurine	0.545 ± 0.132	0.462 ± 0.045	0.329	MWT
Theobromine	0.084 ± 0.017	0.060 ± 0.004	0.084	MWT
Thymine	0.005 ± 0.003	0.002 ± 0.001	0.084	MWT
Thymol	0.121 ± 0.058	0.051 ± 0.004	0.029	MWT
Tiglylglycine	0.003 ± 0.003	0.001 ± 0.001	0.046	MWT
Trigonelline	0.248 ± 0.112	0.096 ± 0.014	0.021	MWT
Trimethylamine	0.009 ± 0.003	0.031 ± 0.009	0.996	MWT

Tyramine	0.000 ± 0.000	0.003 ± 0.002	0.602	MWT
Uracil	0.345 ± 0.180	0.266 ± 0.036	0.987	MWT
Uridine	0.027 ± 0.007	0.024 ± 0.002	0.572	MWT
Valine	0.028 ± 0.005	0.017 ± 0.001	0.029	MWT
Xanthurenate	0.040 ± 0.009	0.021 ± 0.001	0.032	MWT

Table S3. Urinary concentration levels (mmol/L) of metabolites in HTo subgroup and subgroups of hypertensive patients with dyslipidemia (with or without other comorbidities): hypertensive individuals with dyslipidemia only (HTdl), hypertensive individuals with dyslipidemia and diabetes (HTdb•dl) and hypertensive individuals with dyslipidemia and one cardiac disease (HTcd•dl). Data are expressed as "mean ± s.e.m". Statistical analysis was performed using Kruskal-Wallis test (KWT), with *p* value indicated between parenthesis after each metabolite name, followed by post hoc Dunn-Bonferroni Test. * $p < 0.05$ compared with HTo; # $p < 0.05$ compared with HTdl; & $p < 0.05$ compared with HTdb•dl.

METABOLITES	HTo	HTdl	HTdb.dl	HTcd.dl
n	21	22	7	7
D-Galactose (p=0.045)	0.011 ± 0.006	0.009 ± 0.004	0.002 ± 0.002	0.027 ± 0.008*#&
Formate (p=0.044)	0.298 ± 0.078	0.120 ± 0.030*	0.197 ± 0.124	0.097 ± 0.033
Glycine (p=0.007)	0.628 ± 0.084	0.426 ± 0.088*	0.223 ± 0.062**	0.761 ± 0.181&
L-Citramalate (p=0.043)	0.144 ± 0.012	0.127 ± 0.026*	0.094 ± 0.022*	0.158 ± 0.028
Taurine (p=0.045)	0.699 ± 0.116	0.420 ± 0.063	0.191 ± 0.041**	0.528 ± 0.207

Table S4. Urinary concentration levels (mmol/L) of metabolites in HTo subgroup and subgroups of hypertensive patients with diabetes (with or without other comorbidities): hypertensive individuals with diabetes only (HTdb), hypertensive individuals with diabetes and dyslipidemia (HTdb•dl) and hypertensive individuals with diabetes and one cardiac disease (HTcd•db). Data are expressed as "mean ± s.e.m". Statistical analysis was performed using Kruskal-Wallis test (KWT), with *p* value indicated between parenthesis after each metabolite name, followed by post hoc Dunn-Bonferroni Test. * $p < 0.05$ compared with HTo; # $p < 0.05$ compared with HTdb.

METABOLITES	HTo	HTdb	HTdb.dl	HTcd.db
n	21	9	7	5
Glutarate (p=0.021)	0.031 ± 0.006	0.003 ± 0.002**	0.026 ± 0.010	0.028 ± 0.011#
Glycine (p=0.009)	0.628 ± 0.084	0.343 ± 0.146*	0.223 ± 0.062**	0.287 ± 0.136*
Glycolate (p=0.048)	0.507 ± 0.112	0.241 ± 0.041*	0.313 ± 0.074	0.259 ± 0.097*
Methylmalonate (p=0.044)	0.061 ± 0.014	0.043 ± 0.021	0.011 ± 0.006**	0.031 ± 0.012
Taurine (p=0.010)	0.699 ± 0.116	0.255 ± 0.132**	0.191 ± 0.041*	0.347 ± 0.142

Table S5. Urinary concentration levels (mmol/L) of metabolites in HTo subgroup and subgroups of hypertensive patients with one cardiac disease (with or without other comorbidities): hypertensive individuals with one cardiac disease only (HTcd), hypertensive individuals with one cardiac disease and dyslipidemia (HTcd•dl) and hypertensive individuals with one cardiac disease and diabetes (HTcd•db). Data are expressed as "mean ± s.e.m". Statistical analysis was performed using Kruskal-Wallis test (KWT), with P value indicated between parenthesis after each metabolite name, followed by post hoc

Dunn-Bonferroni Test. * $p < 0.05$ compared with HTo; # $p < 0.05$ compared with HTcd; & $p < 0.05$ compared with HTcd•dl.

METABOLITES	HTo	HTcd	HTcd.dl	HTcd.db
n	21	6	7	5
D-Mandelate (p=0.038)	0.0003 ± 0.0000	0.0000 ± 0.0000	0.0000 ± 0.000	0.0028 ± 0.0020*#&
Propylene Glycol (p=0.032)	0.047 ± 0.011	0.016 ± 0.004	0.064 ± 0.029	0.006 ± 0.003*&

Table S6. Receiver operating characteristic (ROC) analysis of acetone, citrate, 4-HPAA, methylglutaconate, N-isovaleroylglycine, oxaloacetate, phenylalanine, pyridoxate, and sarcosine between NT and HTall participants.

METABOLITES	AUC	95% Confidence intervals		Cut-off value	Sensitivity (%)	Specificity (%)
		Lower Bound	Upper Bound			
Acetone	0.792	0.610	0.975	0.015	67.0	88.9
Citrate	0.746	0.553	0.938	2.081	80.0	66.7
4-HPAA	0.766	0.641	0.891	0.062	58.0	88.9
Methylglutaconate	0.771	0.646	0.896	0.046	49.0	100.0
N-Isovaleroylglycine	0.747	0.561	0.933	0.020	89.0	55.6
Oxaloacetate	0.827	0.742	0.912	0.356	73.0	100.0
Phenylalanine	0.730	0.565	0.895	0.085	61.0	88.9
Pyridoxate	0.688	0.517	0.860	0.003	35.0	100.0
Sarcosine	0.682	0.529	0.834	0.007	50.5	88.9

Table S7. Receiver operating characteristic (ROC) analysis of the metabolites that showed significant differences between NT and HTo participants.

METABOLITES	AUC	95% Confidence intervals		Cut-off value	Sensitivity (%)	Specificity (%)
		Lower Bound	Upper Bound			
Acetone	0.778	0.576	0.980	0.015	65.0	88.9
Citrate	0.750	0.532	0.968	2.058	85.0	66.7
4-HPAA	0.753	0.573	0.933	0.062	60.0	88.9
Methylglutaconate	0.797	0.628	0.967	0.044	55.0	100.0
N-Isovaleroylglycine	0.778	0.581	0.974	0.005	75.0	77.8
Oxaloacetate	0.872	0.742	1.002	0.356	80.0	100.0
Phenylalanine	0.744	0.550	0.939	0.083	60.0	88.9
Pyridoxate	0.722	0.528	0.916	0.003	35.0	100.0
Sarcosine	0.761	0.567	0.955	0.007	66.7	88.9
